# Identification of a Protein Arginine Methyltransferase 7 (PRMT7)/Protein Arginine Methyltransferase 9 (PRMT9) Inhibitor

**DOI:** 10.1021/acs.jmedchem.3c01030

**Published:** 2023-08-10

**Authors:** Alessandra Feoli, Giulia Iannelli, Alessandra Cipriano, Ciro Milite, Lei Shen, Zhihao Wang, Andrea Hadjikyriacou, Troy L. Lowe, Cyrus Safaeipour, Monica Viviano, Giuliana Sarno, Elva Morretta, Maria Chiara Monti, Yanzhong Yang, Steven G. Clarke, Sandro Cosconati, Sabrina Castellano, Gianluca Sbardella

**Affiliations:** † Department of Pharmacy, Epigenetic Med Chem Lab, University of Salerno, via Giovanni Paolo II 132, Fisciano ,I-84084 SA Italy; ‡ Department of Pharmacy, ProteoMass Lab, University of Salerno, via Giovanni Paolo II 132, Fisciano ,I-84084 SA Italy; § PhD Program in Drug Discovery and Development, University of Salerno, via Giovanni Paolo II 132, Fisciano ,I-84084 SA Italy; ∥ Department of Chemistry and Biochemistry, and the Molecular Biology Institute, University of California, Los Angeles, California 90095, United States; ⊥ Department of Cancer Genetics and Epigenetics, Beckman Research Institute, City of Hope National Cancer Center, Duarte, California 91010, United States; # DiSTABiF, University of Campania “Luigi Vanvitelli”, Via Vivaldi 43, 81100 Caserta, Italy

## Abstract

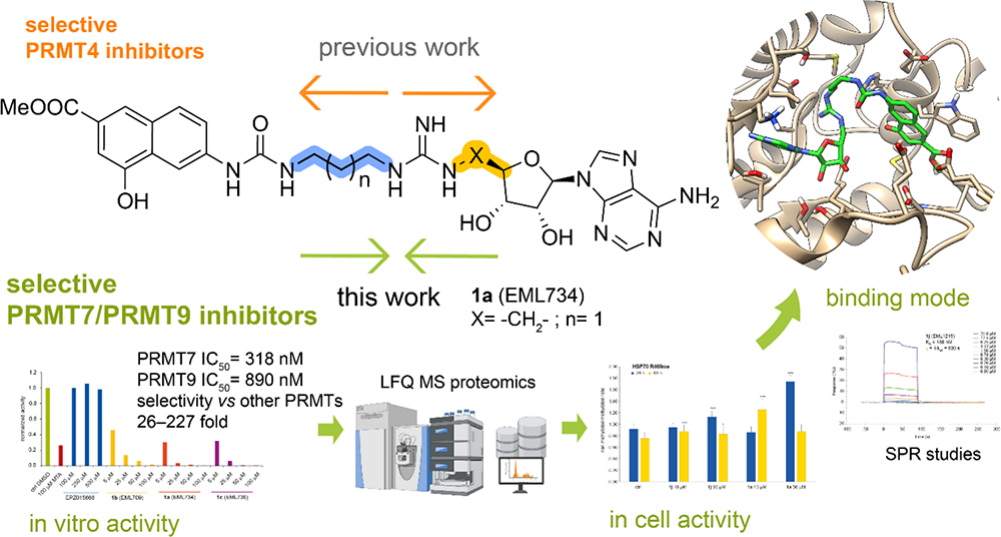

Less studied than the other protein arginine methyltransferase isoforms, PRMT7 and PRMT9 have recently been identified as important therapeutic targets. Yet, most of their biological roles and functions are still to be defined, as well as the structural requirements that could drive the identification of selective modulators of their activity. We recently described the structural requirements that led to the identification of potent and selective PRMT4 inhibitors spanning both the substrate and the cosubstrate pockets. The reanalysis of the data suggested a PRMT7 preferential binding for shorter derivatives and prompted us to extend these structural studies to PRMT9. Here, we report the identification of the first potent PRMT7/9 inhibitor and its binding mode to the two PRMT enzymes. Label-free quantification mass spectrometry confirmed significant inhibition of PRMT activity in cells. We also report the setup of an effective AlphaLISA assay to screen small molecule inhibitors of PRMT9.

## Introduction

Protein arginine methylation has recently attracted growing interest from the scientific community for its role in cell biology and its involvement in physiological and physiopathological processes. As a consequence, the enzymes responsible for this post-translational modification, protein arginine methyltransferases (PRMTs), are increasingly considered promising and relevant targets for drug discovery.^1^

**Figure 1:**
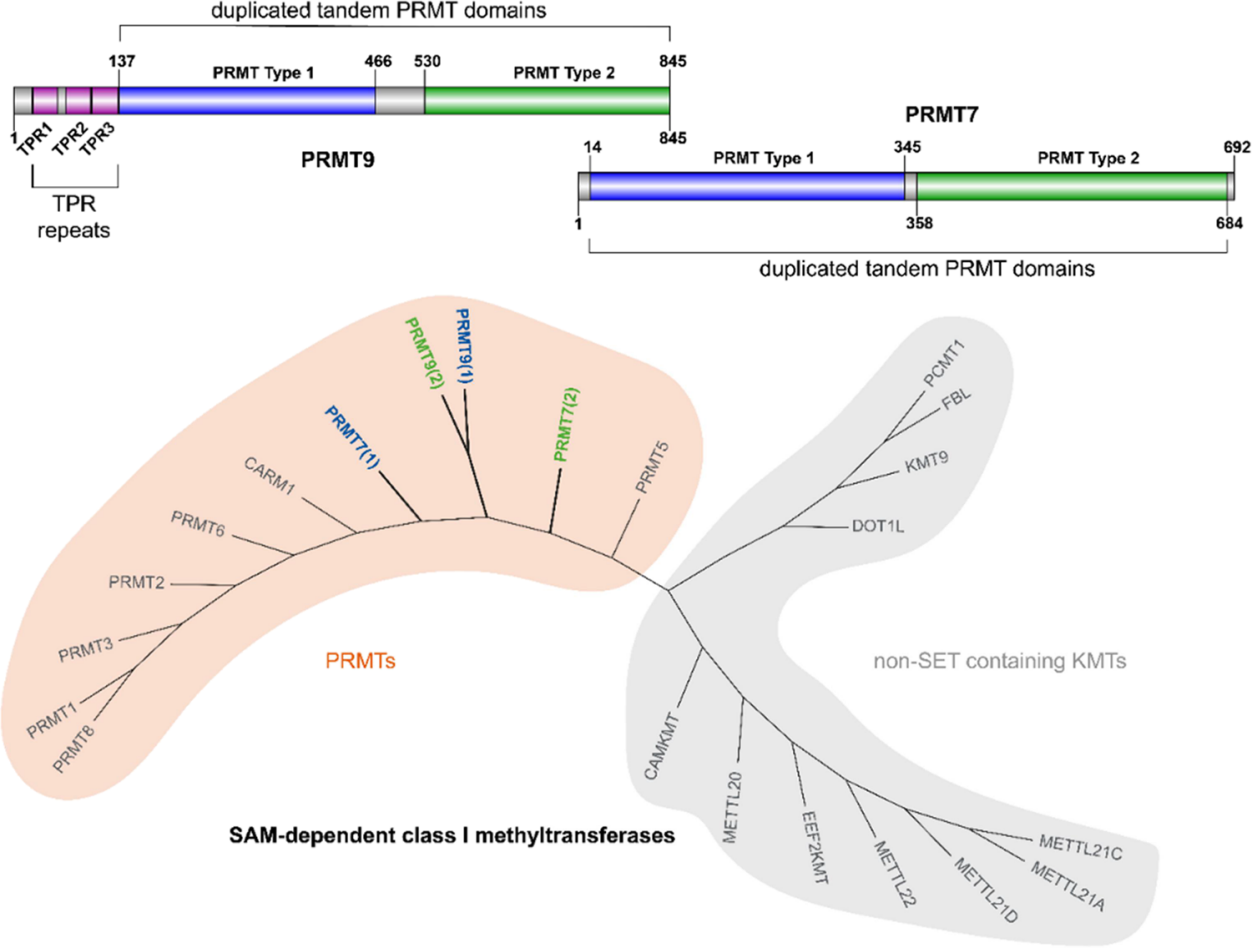
Architecture of PRMT7 and PRMT9 (prepared using Illustrator for Biological Sequences, IBS)^20^ and phylogenetic tree of SAM-dependent class I methyltransferases (obtained with the Structural Genomic Consortium ChromoHub^21^ and modified with Adobe Illustrator CC 2023). PRMTs are highlighted in the orange area, whereas non-SET domain-containing KMTs are in the gray area.

In mammals, nine sequence-related PRMT isoforms (PRMT1–PRMT9) have been identified, and they are classified into three subfamilies (Type I, Type II, and Type III) based on the product of the methylation reaction they catalyze. In particular, type I PRMTs (PRMT1, PRMT2, PRMT3, PRMT4, PRMT6, and PRMT8) catalyze the formation of monomethylarginine (Rme1)^2^ and asymmetric dimethylarginine (Rme2a), type II PRMTs (PRMT5 and PRMT9) catalyze the formation of Rme1 and symmetric dimethylarginine (Rme2s), whereas type III PRMT (PRMT7) catalyzes only the formation of Rme1.^3,4^ The nine members of the PRMT family share a common Rossmann-like fold seven-stranded β-sheet connected by α-helices and a β-barrel domain. They have been classified as class I *S*-adenosylmethionine (SAM)-dependent methyltransferases, together with some non-SET domain lysine methyltransferases (e.g., DOT1L; Figure 1) also featuring the same elements.^5,6^

Several PRMT inhibitors have been identified to date, mostly type I PRMT inhibitors, both selective and unselective, and PRMT5 inhibitors.^1,7,8^ Only recently the first PRMT7 selective inhibitor, SGC8158, was reported,^9^ while no inhibitor for PRMT9 has been described so far.^10^ However, very little is known about these two methyltransferases. While all the other PRMTs contain only one methyltransferase domain, both PRMT7 and PRMT9 contain two tandem domains resulting from ancestral duplication (Figure 1). The single C-terminal domain is catalytically inactive, yet it is necessary for enzyme activity being folded together with the N-terminal one to form a pseudodimer.^11−13,14,15,16,17^ PRMT7 is associated with metastasis and DNA damage and is considered a potential target for treating breast cancer,^18,19^ but important questions regarding its major role in cell biology are still open since it does not prime for dimethylation by type I and type II PRMTs.^15^

Seemingly, most of the biological roles of PRMT9 remain to be further defined along with its substrates.^13^

Nonetheless, PRMT9 has been identified as a potential target for treating hepatocellular carcinoma,^22,23^ for suppressing acute myeloid leukemia maintenance,^24^ and it is required for androgen-dependent proliferation of LNCaP prostate cancer cells.^25^ It has been reported to play a role in the regulation of alternative splicing.^11,13^ Very recently, it has been shown that PRMT9 attenuates activation of mitochondrial antiviral signaling protein (MAVS) through arginine methylation (R41 and R43), thus reducing innate antiviral immune response.^26^

Pursuing our interest in the identification of potent and selective PRMT inhibitors,^27−29,30,31,32,33,34,35,36,37,38^ we were intrigued by the identification of hits for the development of PRMT9 inhibitors.

Identification of potent and selective PRMT9 inhibitors is a challenge due to several factors. Crystal structures of PRMTs have provided extensive amounts of information to aid in the development of selective inhibitors^1^ but only recently a crystal structure of PRMT9 has become available (PDB ID: 6PDM),^39^ and no literature report has been published up to date. In addition, the discovery of PRMT inhibitors requires efficient and effective biochemical screening assays for measuring their methyltransferase activity. However, in the case of PRMT9, there are only a limited number of screening techniques,^8^ and no commercially available antibody can recognize R508me2s resulting from the enzymatic activity of PRMT9 on its specific substrate splicing factor 3B subunit 2 (SF3B2, also known as spliceosome-associated protein 145 SAP145).^13^

Overall, although many potent small molecule inhibitors have been reported for PRMTs, selectivity remains a challenge for individual PRMT isoforms because of a conserved SAM binding site and similar substrate recognition motif. On the other hand, even subtle modifications of chemical structure can greatly influence selectivity, and even close analogs of SAM can be surprisingly selective. Moreover, several lines of evidence support the essential role of the distance between the pharmacophore groups in PRMTs inhibition potency and selectivity.^31,40−42,43,44^

In SGC8158, for instance, changing the methylene linker length between the terminal amine moiety and the adenosine core resulted in a decrease in selectivity.^9^

In a recent study, we demonstrated that modulating the distance between pharmacophoric moieties leads to potent and selective PRMT4 inhibitors.^31^ On the contrary, PRMT7 seems to preferentially bind derivatives with shorter linkers.

Based on the abovementioned considerations, we resolved to extend these structural studies to PRMT9 and to further explore the features of the binding of compounds spanning both the substrate and the cosubstrate pockets for the further development of inhibitors.

Herein, we report the identification of the first potent PRMT7/9 inhibitor and its binding mode to the two PRMT enzymes. We also report the setup of an effective assay to screen small molecule inhibitors of PRMT9.

## Results and Discussion

### Optimization of Alpha-Based Screening for PRMT9 Inhibitor Identification

To date, radiometric assays represent the only standard for biochemically measuring the methyltransferase activity of PRMT9. Yet, the cost, the difficulty to automate, and the danger associated with their usage and the generation of radioactive waste are huge deterrents for random or target-based screening. Recently, a PRMT9 homogeneous assay kit in an AlphaLISA format has become commercially available (BPS Bioscience #52069) and, even if, to the best of our knowledge, no evidence of its effectiveness in small molecules screening has been reported in the literature so far,^45^ at first we decided to use it for the evaluation of the inhibitory effect of our compounds against PRMT9. The assay was based on PRMT9-mediated methylation of the GST-tagged substrate (an unspecified SF3B2 peptide) in the presence of the SAM cosubstrate. The modification resulting from the enzymatic reaction was detected by adding a primary antibody (specifically recognizing the substrate methylated arginine), antirabbit IgG acceptor beads (capturing the Fc region of the primary antibody), and glutathione donor beads (capturing the GST tag of the substrate).

Unfortunately, following the protocol reported for this assay, we obtained low alpha counts and an unacceptable signal/noise ratio, with a blank higher than the controls (Figure S1A, Supporting Information). We assumed that this unexpected behavior could be due to a nonspecific recognition of the substrate and/or to an interference from the GST tag of the substrate. Accordingly, we tried to optimize the assay by diluting the substrate 1:10, but we did not observe any improvement (Figure S1B). We reported the issue to the BPS Technical Support, which suggested an 8-fold increase in the enzyme amount to obtain a signal/noise ratio of 2.3. However, this was still unacceptable and also resulted in the need for significantly larger amounts of enzyme. Therefore, we redesigned the assay using a biotinylated 20-amino acid peptide of SF3B2 (aa 500–519) instead of the GST-substrate of the kit and streptavidin donor beads instead of glutathione ones. Thanks to these modifications, we were finally able to obtain a good signal/noise ratio, avoiding the previously reported problems with blanks (Figure S1C). Accordingly, all the enzymatic inhibition data were obtained by performing the assays in these optimized conditions. Noteworthy, while writing this manuscript, we were pleased to find out that BPS changed the kit, replacing the GST-tagged substrate with a biotinylated one and including a 5-fold higher amount of the enzyme (with a higher level of purity), substantially confirming our modifications to the assay.^46^

**Figure 2:**
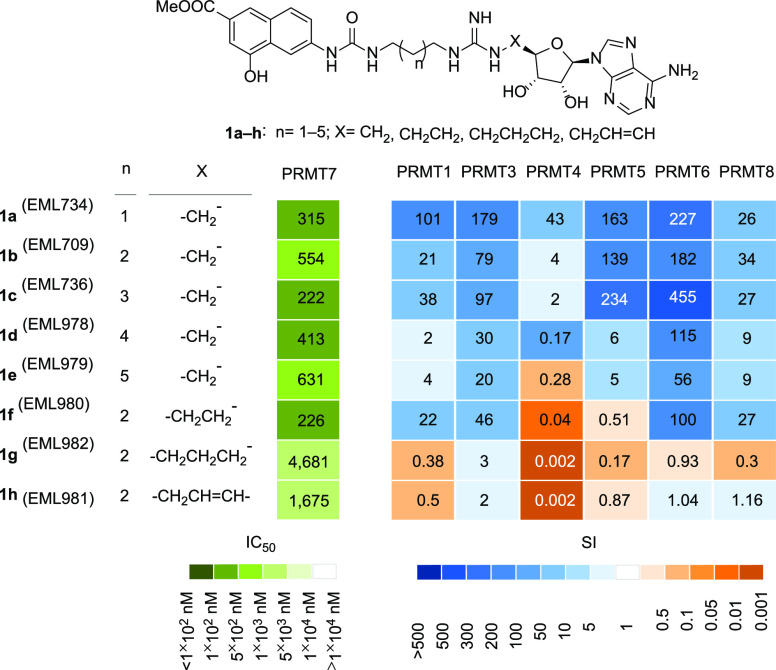
Inhibitory activities of compounds **1a**–**h** against PRMT7:^31^ the heatmaps depict the IC_50_ values (nM) for compounds **1a**–**1h** against PRMT7 (left panel, in shades of green) and the selectivity index (fold) for PRMT7 over the specified PRMT (right, shades of blue and orange).

Recently, we successfully applied a deconstruction–reconstruction and fragment-growing approach to achieve potency and selectivity against PRMT4 starting from nonselective PRMT inhibitors. This approach allowed us to investigate the structural features of the binding to PRMT enzymes and we found that the overall length of synthesized compounds **1a**–**h** (Figure 2) and, even more, the length of the linkers between the 4-hydroxy-2-naphthoate-6-urea and the guanidine group and between the latter and the adenosine moiety resulted to be crucial for the inhibitory activity especially against PRMT4 and, at a minor extent, against PRMT1.^31^ On the contrary, the study showed that increasing the linker length is detrimental for the inhibition of PRMT6 because the resulting compounds are forced to adopt an odd distorted U-shaped conformation that reduces the favorable binding interactions with the enzyme double-E loop clamp and the arginine substrate pocket.^31^ Interestingly, we observed a different trend for the inhibitory activity against PRMT7, with the shorter compound (**1a**, EML 734) showing an IC_50_ value of 0.32 μM and a certain selectivity compared to other tested PRMTs (SI values in the range 26–227; see Figure 2).

**Figure 3:**
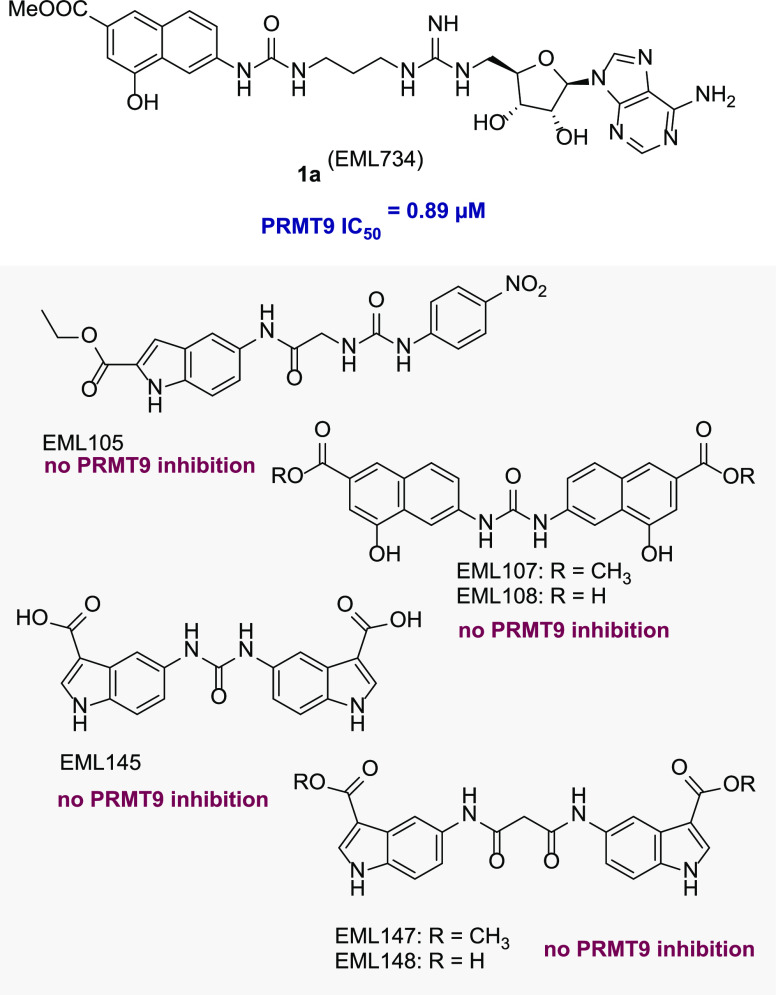
Inhibitory activities of selected PRMT modulators (from in-house libraries) against PRMT9.

This is consistent with the restrictive and narrow active site described for PRMT7.^15,47^ Therefore, we decided to start our structural studies on PRMT9 by investigating the capability of **1a** (EML734) to inhibit the enzyme in our AlphaLISA assay. For comparison, we also selected a few compounds from our in-house libraries of PRMT modulators^27,28,31,35^ among those featuring some structural similarities with **1a** (e.g., the hydroxy-carboxy-naphthyl-urea portion, or the bioisosteric carboxy-indolyl-urea or carboxy-indolyl-amide moieties) and tested them for their ability to inhibit PRMT9 (Figure 3).

### Selection of Compounds for Preliminary Screening and Hit Identification

Both bis-4-hydroxy-2-naphthoic compounds (EML107 and EML108)^27^ and bis-indolecarboxylate compounds (EML145, EML147, and EML148),^28^ previously identified as class I PRMT inhibitors, as well as the aryl acetamido ureido indole carboxylate (“uracandolate”) EML105 (an enhancer of PRMT4 activity),^28^ showed no activity against PRMT9. On the contrary, compound **1a** exhibited good inhibitory activity against PRMT9, with a submicromolar IC_50_ value (0.89 μM).

Prompted by this result, we turned back our attention to the derivatives of **1a** previously synthesized by us (compounds **1b**–**h**) and tested them in our in-house AlphaLISA assay, with the aim of investigating the effect of the modulation of the distance between the pharmacophoric moieties on the inhibiting activity against PRMT9.

We found that in the case of PRMT9, the distance between the methyl 4-hydroxy-2-naphthoate moiety and the arginine-mimetic group does not significantly affect the inhibitory activity of the compounds, with all the compounds **1a**–**e** (*n*= 1–5; Table 1) showing comparable and relatively good inhibiting properties (IC_50_ values around 1 μM) against PRMT9. A decrease in the inhibitory activity was observed when the linker between the guanidine group and the adenosine moiety was more than two-carbon atoms long. In fact, compounds **1g** and **1h** were the least effective inhibitors, with an opposite trend with respect to what we previously observed for PRMT4.^31^ Compound **1a**, featuring a propyl spacer between the 4-hydroxy-2-naphthoate moiety and the guanidine group, was the most potent derivative with a submicromolar activity against PRMT9 and a good selectivity profile against the other PRMTs.

**Table 1:**
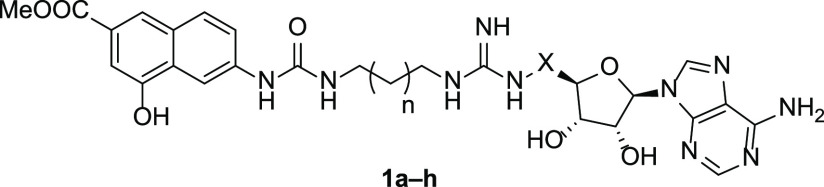
Inhibitory Activities of Compounds **1a–h** against PRMT9

compound	*n*	X	PRMT9 IC_50_^a^^,^^b^ (μM)
**1a** (EML734)	1	–CH_2_–	0.89
**1b** (EML709)	2	–CH_2_–	1.32
**1c** (EML736)	3	–CH_2_–	5.80
**1d** (EML978)	4	–CH_2_–	1.02
**1e** (EML979)	5	–CH_2_–	1.30
**1f** (EML980)	2	–CH_2_–CH_2_–	1.20
**1g** (EML982)	2	–CH_2_–CH_2_–CH_2_–	>10.00
**1h** (EML981)	2	–CH_2_–CH=CH–	9.30

a Obtained in AlphaLISA assay, using human recombinant PRMT9 (0.105 μM, final concentration). SF3B2 (500–519) peptide, biotinylated (100 nM, final concentration), and SAM (25 μM, final concentration) were used as substrate and cosubstrate, respectively.

b Compounds were tested in 10-concentration IC_50_ mode with 3-fold serial dilutions starting at 100 μM. Data were analyzed with GraphPad Prism software (version 6.0) for IC_50_ curve fitting.

Design, synthesis, and inhibitory activity of **1i** (EML1102), the lower homologue of **1a** (EML734).

**Figure 4:**
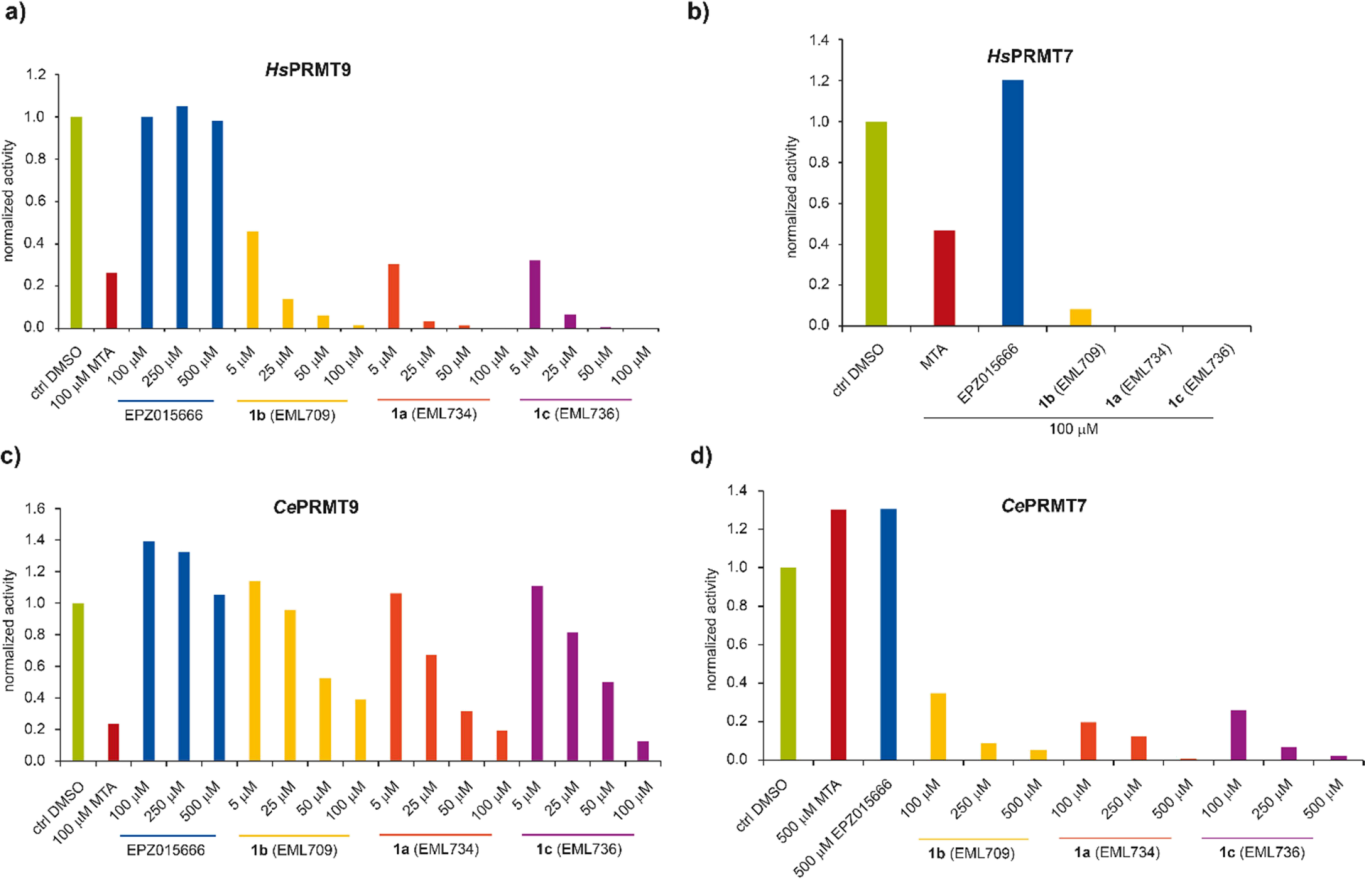
Inhibition of GST-tagged *Hs*PRMT9 and *Hs*PRMT7 (panels a and b, respectively) or *Ce*PRMT7 and *Ce*PRMT9 (panels c and d, respectively) by compounds **1a**–**c** as detected by a radioisotope-based assay. The experiments were performed as reported in the Experimental procedures section. GST-*Hs*PRMT9 (a) and GST-*Hs*PRMT7 (b) were incubated with human GST-SF3B2 (401–550) peptide or recombinant *Hs*H2B, respectively (1 μg of enzyme, 5 μg of substrate), 0.14 μM [^3^H]SAM, and the indicated concentrations of tested compounds at the corresponding optimal reaction temperature (37 °C for *Hs*PRMT9, 15 °C for *Hs*PRMT7). *C. elegans* GST-tagged enzymes PRMT9 (c) and PRMT7 (d) were incubated with GST-*Ce*SFTB-2 (99–248) fragment or recombinant *Hs*H2B, respectively (1 μg of enzyme, 5 μg of substrate), 0.14 μM [^3^H]SAM, and the indicated concentrations of tested compounds at the corresponding optimal reaction temperature (25 °C for *Ce*PRMT9, 15 °C for *Ce*PRMT7). After SDS-PAGE, the gels were treated as previously described^48^ and densitometry analysis was done using ImageJ software, and data was plotted as normalized activity to the no inhibitor controls.

Based on these outcomes, we resolved to explore the effect on the capability to inhibit PRMT9 enzymatic activity of further reduction of the distance between the pharmacophoric moieties. Therefore, we synthesized compound **1i** (EML1102) in which the propyl spacer of **1a** was replaced with the shorter ethyl group and tested it against PRMT9 as well as against all the other PRMTs (with the only exception of PRMT2; Table 2). As shown in Table 2, compound **1i** confirmed the general trend previously observed for compound **1a**–**h** against type I PRMTs. In fact, the reduction of the spacer length further reduced the inhibitory potency against PRMT1, PRMT3, PRMT6, and PRMT8 (compare the activities of **1i** and **1a** in Table 2). Consistent with the geometric restriction of the enzyme active site,^15,47^ compound **1i** substantially maintained the inhibitory activity of **1a** against PRMT7. On the contrary, the further reduction of the length of the alkyl spacer between the 4-hydroxy-2-naphthoate moiety and the guanidine group resulted in being detrimental to the inhibition of PRMT9. In fact, as reported in Table 2, compound **1i** exhibited a 3-fold reduction in potency compared to its next higher homologue **1a**. To confirm the activity of the compounds against PRMT9, we then used a radioisotope-based assay as a secondary screening approach.^48,49^ In these experiments, *Hs*PRMT9 was incubated with ^3^H-SAM and SF3B2 (401–550) peptide with and without inhibitors **1a**–**c** at the reported concentrations, and then the reaction products were analyzed by SDS gel electrophoresis followed by fluorography and densitometric analysis. 5′-Deoxy-5′-(methylthio)adenosine (methylthioadenosine, MTA), the polyamine byproduct in the methionine salvage pathway that is reported to be a SAM-competitive inhibitor of PRMTs,^50−52,53,54^ and the PRMT5 inhibitor EPZ015666 (GSK3235025)^50,55^ were used as reference drugs. As shown in Figure 4a and consistent with the results of the AlphaLISA assay, all three compounds induced half inhibition of PRMT9 at a concentration lower than 5 μM. The effect is concentration-dependent, and almost complete inhibition was observed at 100 μM. On the contrary, MTA was able to give a good inhibition only at 100 μM, whereas EPZ015666 was inactive up to 500 μM. For comparison, compounds **1a**–**c** were tested in the same assay also against *Hs*PRMT7, in the presence of recombinant *Hs*H2B as a substrate (Figure 4b). As expected, the inhibiting activities were consistent with those reported in Figure 2. Both PRMT7 and PRMT9 are evolutionarily conserved proteins, with distinct orthologs in plants, invertebrates, and vertebrates, and human enzymes have much in common with their orthologs from the soil nematode worm *Caenorhabditis elegans*,^48^ which has developed into an important model for the functional characterization of various drug targets,^56^ including PRMTs.^57^ Nonetheless, important differences in terms of active site architecture and substrate specificity have been reported between human and nematode PRMT7 proteins, whereas the two PRMT9 orthologs appear to be biochemically indistinguishable.^48^ Therefore, we decided to investigate the effects of compounds **1a**–**c** also on the enzymes from *C. elegans*. A nearly full inhibition of *Ce*PRMT9 was observed with each of the three inhibitors at 100 μM concentration, while the half inhibition was observed between 25 and 50 μM, suggesting that the inhibitors have maybe 5–10-fold less binding affinity for the worm enzyme than for the human enzyme (Figure 4c). On the contrary, consistent with the previously reported differences, not much inhibition was seen with the *Ce*PRMT7 enzyme using histone H2B as a substrate at 100 μM concentrations. After the experiment was repeated with 100, 250, and 500 μM concentrations of each inhibitor, half inhibition was observed only at a concentration of roughly 250 μM (Figure 4d).

**Table 2:**
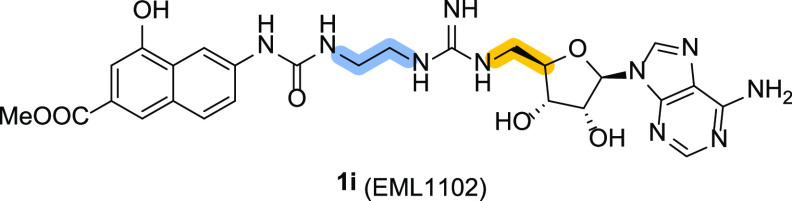
Inhibitory Activities of Compound **1i** against PRMTs

	IC_50_^a^^,^^b^ (μM)							
cmpd	PRMT1	PRMT3	PRMT4	PRMT5	PRMT6	PRMT7	PRMT8	PRMT9
**1a** (EML734)	32.27^c^	57.19^c^	13.84^c^	52.13^c^	72.77^c^	0.32^c^	8.29^c^	0.89^d^
**1i** (EML1102)	>100	>100	22.8	9.4	70	0.54	42	2.47^d^

a Compounds were tested in 10-concentration IC_50_ mode with 3-fold serial dilutions starting at 100 μM. Data were analyzed with GraphPad Prism software (version 6.0) for IC_50_ curve fitting.

b Unless differently indicated, the values were obtained in a radioisotope-based filter assay, using 5 μM histone H4 (for PRMT1, PRMT3, and PRMT8), histone H3 (for PRMT4), histone H2A (for PRMT5), or GST-GAR (for PRMT6 and PRMT7) as the substrate and *S*-adenosyl-l-[methyl-^3^H]methionine (1 μM) as a methyl donor.

c Data from ref (31).

d Obtained in AlphaLISA assay, using human recombinant PRMT9 (0.105 μM, final concentration). SF3B2 (500–519) peptide, biotinylated (100 nM, final concentration) and SAM (25 μM, final concentration) were used as substrate and cosubstrate, respectively.

### Docking and Structure-Based Ligand Design Studies

**Figure 5:**
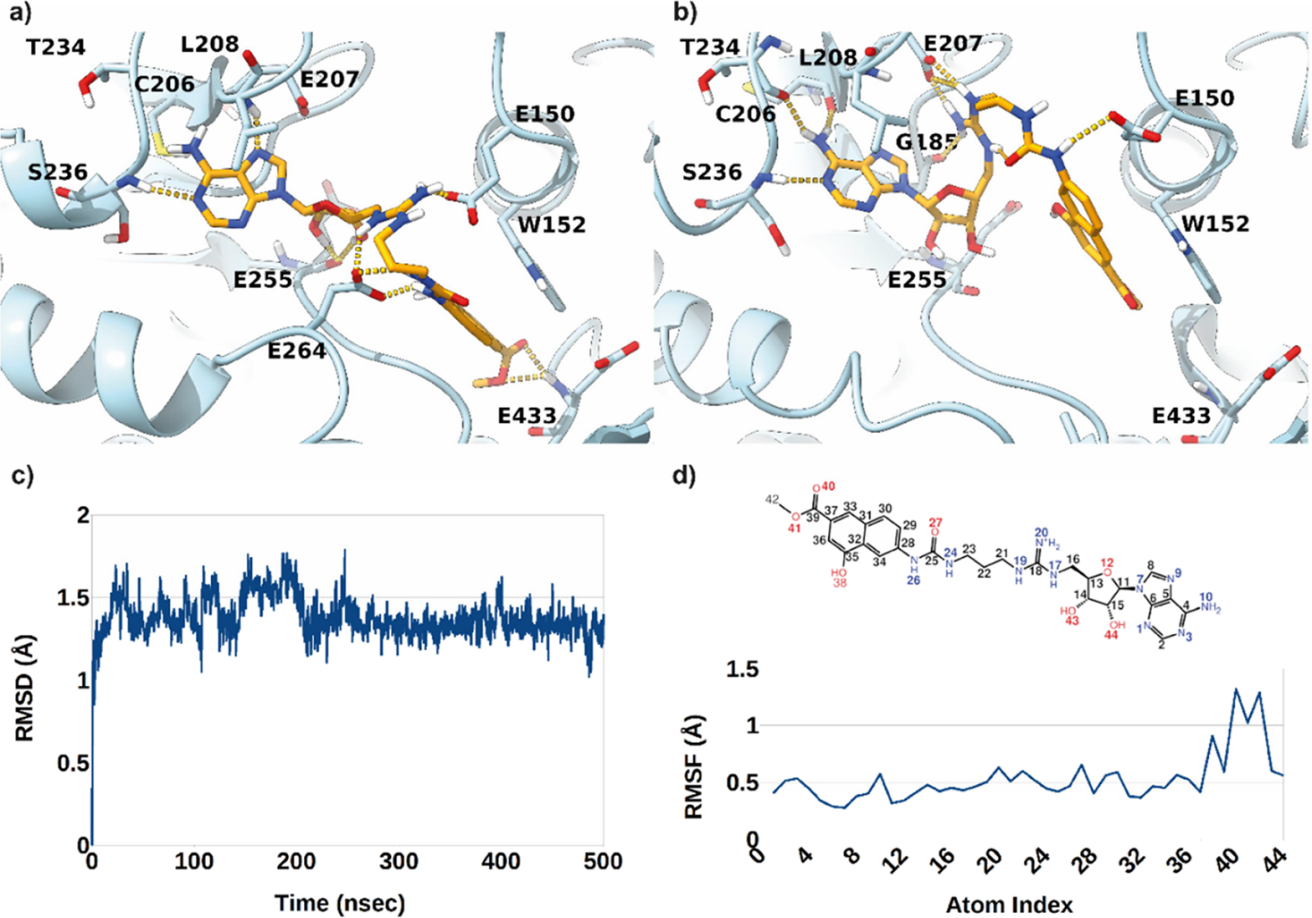
Binding mode of **1a** in complex with the PRMT9 3D structure (PDB entry 7RBQ) as predicted by docking calculations (a) and representative frame of the 500 ns long MD simulation (b). The ligand and enzyme are represented as orange and cyan stick and ribbons, respectively. L-RMSD (c) and L-RMSF (d) plots obtained from the analysis of MD simulations.

Prompted by the results of preliminary structure–activity relationship (SAR) studies, molecular modeling calculations were attempted to propose a viable binding interaction model between our most potent and sufficiently selective PRMT9 ligand, **1a** (EML734), and the enzyme and prospectively suggest possible modifications that could enhance the ligand/enzyme recognition. In particular, **1a** was subjected to docking calculations employing the latest OpenCL implementation of AutoDock4, called AutoDock-GPU (AD4-GPU),^58^ and the recently released cocrystal structure of the human PRMT9 in complex with another adenosine-based inhibitor (MT556, PDB code 7RBQ).^59^ Results of docking analysis revealed that in the predicted lowest-energy binding orientation (Δ*G*_AD4_ = −14.4 kcal/mol), the ligand adenine ring is able to occupy the protein region engaged by the same ring in the cocrystal ligand. Here, H-bond interactions are established with the L208 and S236 backbone NHs groups. Additional H-bonds are formed by the sugar OH groups with the E255 backbone CO, while the arginine-mimetic group is involved in ionic contacts with the E150 and E264 negatively charged side chains. This latter residue is also involved in charged-reinforced hydrogen bonds with the ligand urea moiety. Finally, the 4-hydroxy-2-naphthoate group is inserted in a rather lipophilic cleft establishing a π–π interaction with the W152 side chain, while the methyl ester in position 6 is H-bonding E433 backbone NH. On the contrary, no specific contacts were predicted for the 4-OH group of the naphthyl ring. To probe the stability of the interactions predicted by AD4 as well as include the effect of the solvent in mediating the ligand/protein contacts, the above-described **1a**/PRMT9 complex was subjected to a 500 ns long molecular dynamics simulations employing the Desmond MD software.^60^ Analysis of the achieved results demonstrated that the predicted binding pose is fairly stable over the simulation time, as demonstrated by the ligand root-mean-square deviations and fluctuations plots (L-RMSD and L-RMSF, respectively, Figure 5c,d). Indeed, the average RMSD value is fairly low (1.38 Å with a standard deviation of 0.12) with the most flexible part residing in the ligand naphthyl tail which experiences a partial relocation probably induced by the flexible linking spacer between this ring and the adenine one. This relocation is made possible by a rather stable intramolecular charge-reinforced H-bond established by the urea carbonyl oxygen and the positively charged guanidinium group. While relocating the terminal moieties of the ligand are still able to establish the same sort of interactions with the protein counterpart. In particular, the adenine ring engages H-bonds with C206, T234, and S236 backbone atoms while the naphthyl ring π-stacking contacts with W152. While this latter contact seems to be stable throughout the simulation, the naphthyl hydroxy and methyl ester substituents do not seem to have direct and stable interactions with PRMT9. Compound **1a** is demonstrated to be a proficient PRMT7 inhibitor. Therefore, molecular modeling studies were also attempted on this latter enzyme. Unfortunately, up to date no experimental structure of the human PRMT7 enzyme has been reported, while the structure of the murine orthologue has been solved in complex with the SGC8158 chemical probe. We resolved to analyze the structure of the human protein as calculated by AlphaFold^61^ and compared it to the mouse one, demonstrating no substantial difference in the overall folding (data not shown). Therefore, also considering that the activity data were obtained using the human PRMT7, in this inspection, we decided to utilize the human protein. In particular, the same protocol of docking + MD simulations used for PRMT9 was employed, demonstrating a comparable interaction pattern. More precisely, as happened for the predicted binding pose in the human PRMT9, also for the PRMT7 enzyme, the ligand adenosine and sugar rings are involved in H-bond interactions reminiscent of the interactions established by the same rings in the SAM cosubstrate.

**Figure 6:**
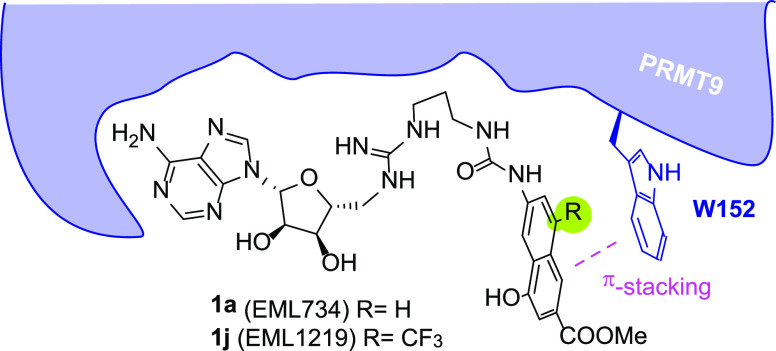
Design of compound **1j** to strengthen π-stacking interaction with the PRMT9 W152 residue (in blue).

Additionally, the guanidine portion is involved in ionic contacts with negatively charged residues. The main differences, however, are recorded for the terminal 4-hydroxy-2-naphthoate group that in this case is pointing toward a rather solvent-exposed and hydrophilic protein region. This set of interactions is well preserved throughout the entire 500 ns MD simulation (Figure S2, Supporting Information). To further validate the presented ligand/PRMT9 interaction model, we decided to design an analogue of **1a** capable of further enhancing recognition with this latter enzyme. Specifically, we wanted to reinforce the contact with W152 of the naphthyl ring by decorating it with an electron-withdrawing group capable of strengthening the π–π contact with this residue. We decided to synthesize compound **1j** (EML1219; Figure 6) featuring a trifluoromethyl group at position 8 of the above-mentioned ring. This position was selected because, being solvent exposed, no unwanted steric clash with the enzyme binding site was expected.

To characterize the effect of compound **1j** on PRMT9, we resolved to evaluate its direct binding to the target protein using surface plasmon resonance (SPR). To this aim, human recombinant PRMT9 (2–845; N-terminal FLAG-tag, C-terminal His6-tag) was covalently immobilized on a sensor chip surface using an amine coupling approach and compound **1j** was injected over the active and reference cells at 10 different concentrations (2-fold dilution series) from 25 to 0.05 μM using the multicycle modality. Each injection was performed with an association and a dissociation time of 90 and 180 s, respectively, and with a flow rate of 30 μL/min. To reduce false positives from detergent-sensitive, nonspecific aggregation-based binding, detergents (0.05% Tween20) were added to the running buffer in all experiments. The corrected sensorgrams were fitted simultaneously by kinetic analysis using the 1:1 Langmuir model of the BIAevaluation software to obtain equilibrium dissociation constants (*K*_D_) and kinetic dissociation (*k*_off_) and association (*k*_on_) constants, and the curve-fitting efficiency was evaluated by chi-square (χ^2^). The χ^2^ value of **1j** was calculated to be 0.650, indicating a good fit.

**Figure 7:**
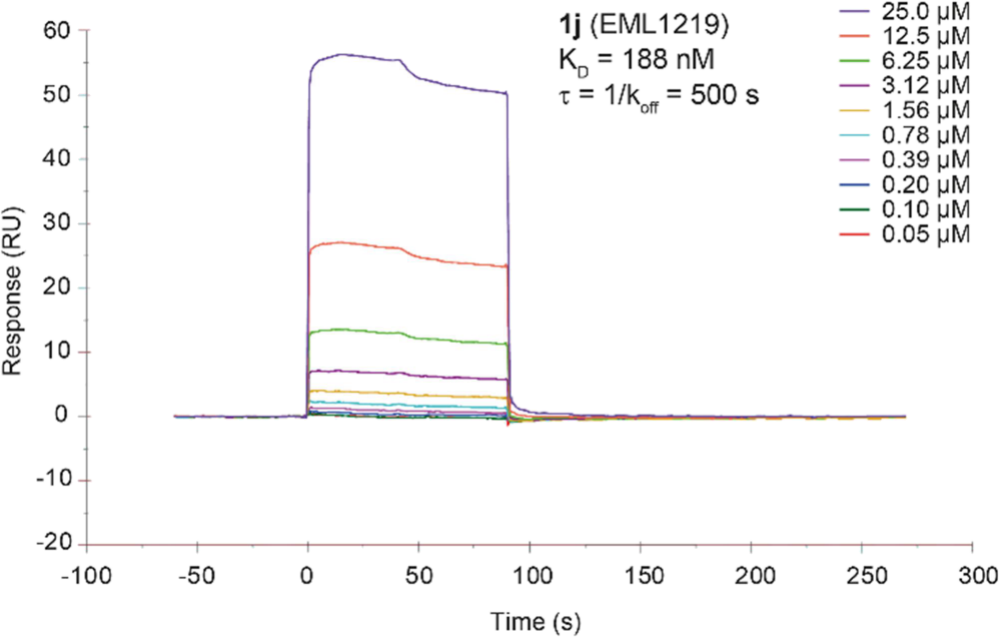
Sensorgrams obtained from the SPR interaction analysis of compound **1j** binding to immobilized PRMT9. The compound was injected at different concentrations (from 25 to 0.05 mM) with an association and a dissociation time of 90 and 180 s, respectively, and with a flow rate of 30 μL/min. The equilibrium dissociation constant (*K*_D_) was derived from the ratio between kinetic dissociation (*k*_off_) and association (*k*_on_) constants.

SPR studies demonstrated a specific and strong binding interaction between PRMT9 and the compound, with an equilibrium dissociation constant (*K*_D_) value in the submicromolar range (*K*_D_ = 188 nM; Figure 7) and a rather high in vitro residence time value (τ_R_ = 500 s).

Next, we tested **1j** against PRMT9 as well as the other PRMTs (with the only exception being PRMT2; Table 3).

**Table 3:**
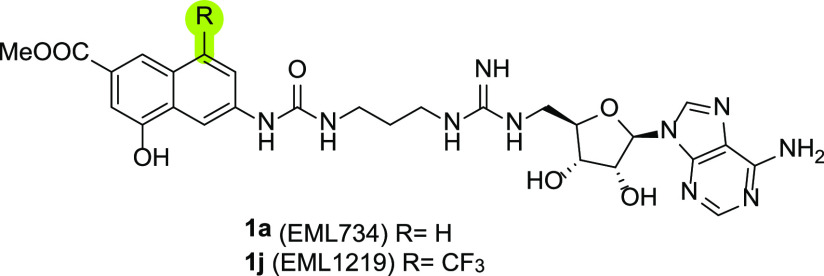
Inhibitory Activities of Compound **1j** against PRMTs

	IC_50_^a^^,^^b^ (μM)							
cmpd	PRMT1	PRMT3	PRMT4	PRMT5	PRMT6	PRMT7	PRMT8	PRMT9
**1a** (EML734)	32.27^c^	57.19^c^	13.84^c^	52.13^c^	72.77^c^	0.32^c^	8.29^c^	0.89^d^
**1j** (EML1219)	48.9	14.9	1.46	1.01	49.7	5.6	1.97	0.2^d^

a Compounds were tested in 10-concentration IC_50_ mode with 3-fold serial dilutions starting at 100 μM. Data were analyzed with GraphPad Prism software (version 6.0) for IC_50_ curve fitting.

b Unless differently indicated, the values were obtained in a radioisotope-based filter assay, using 5 μM histone H4 (for PRMT1, PRMT3, and PRMT8), histone H3 (for PRMT4), histone H2A (for PRMT5) or GST-GAR (for PRMT6 and PRMT7) as substrate and *S*-adenosyl-l-[methyl-^3^H]methionine (1 μM) as the methyl donor.

c Data from ref (31).

d Obtained in AlphaLISA assay, using human recombinant PRMT9 (0.105 μM, final concentration). SF3B2 (500–519) peptide, biotinylated (100 nM, final concentration), and SAM (25 μM, final concentration) were used as substrate and cosubstrate, respectively.

As shown in Table 3, the introduction of the trifluoromethyl substituent at position 8 of the naphthyl ring resulted in a 4-fold increased inhibitory potency against PRMT9, thus confirming the predicted binding mode. However, the gain in target affinity comes at a cost in selectivity, particularly against the other type II enzyme, PRMT5 (Table 3).

The selectivity of compound **1j** was further assessed against a panel of eight lysine methyltransferases (KMTs), including the SET-domain-containing proteins ASH1L/KMT2H, EZH2/KMT6 (5 component complex), G9a/KMT1C, MLL1/KMT2A (5 component complex), SET7/9/KMT7, SMYD3/KMT3E, SUV39H2/KMT1B, and the non-SET domain containing DOT1L/KMT4.^6^ To this aim, the inhibition of **1j** toward these selected enzymes was assessed at two different concentrations (10 and 100 μM, respectively, >50 and >500 fold higher than the IC_50_ value against PRMT9) using SAH,^62−64^ or chaetocin (for ASH1L)^65^ as reference compounds. Noteworthy, we found that none of the SET-domain-containing enzymes was inhibited by **1j** even at the highest tested concentration (Figure S3 and Table S1, Supporting Information), whereas the non-SET domain containing DOT1L/KMT4 was significantly inhibited even at 10 μM, thus confirming that the introduction of the trifluoromethyl group gives a reduction in selectivity within class I SAM-dependent methyltransferases.

### Assessment of in Cell Functional Potency

As mentioned above, compounds **1a**–**h** were originally designed to probe the structural differences among the various PRMTs in order to gain important information for the development of potent and selective inhibitors and nonoptimized for cell permeability. Yet, we previously reported that, regardless of its low cell permeability, **1h** is able to induce an evident reduction of PRMT4-catalyzed arginine methylation levels in MCF-7 cells and a marked reduction of proliferation.^31^

Therefore, we resolved to investigate whether the compounds can reduce the cellular level of arginine methylation catalyzed by PRMT9. Note that when SF3B2 was characterized as the methylation substrate of PRMT9, a homemade methyl-specific antibody was developed to detect PRMT9-catalyzed SF3B2 methylation site (R508), namely, SF3B2 R508me2s.^13^ This antibody was validated as very specific and not affected by the low reproducibility issues that often plague many antibodies used for detection of PTMs.^66,67^ To test the effect of compounds **1a**, **1c**, **1e**, and **1f** on PRMT9 activity in vivo, we treated MCF-7 and MDA-MB-436 breast cancer cell lines with these compounds at indicated concentrations for 72 h, and the total cell lysates were then immunoblotted with the αSF3B2 R508me2s methyl-specific antibody.

**Figure 8:**
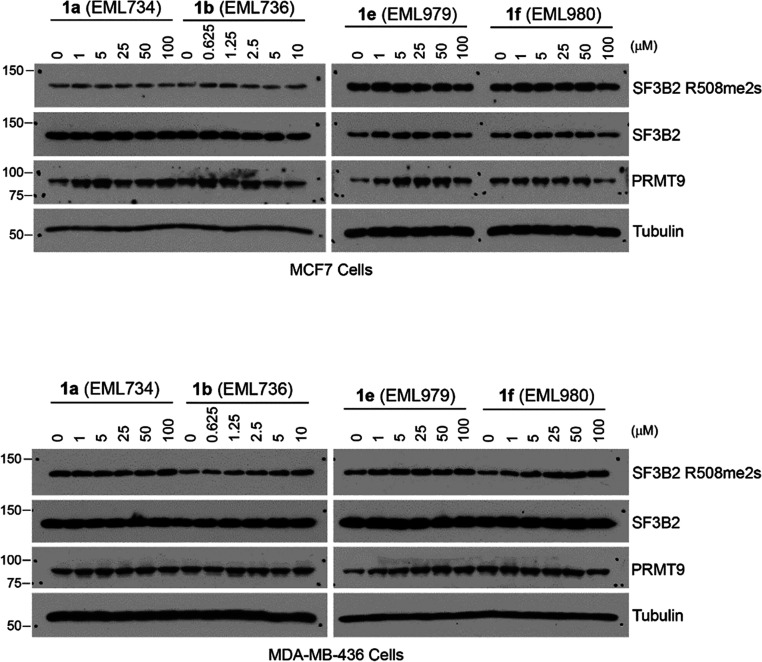
Testing the effects of compounds **1a** (EML734), **1b** (EML736), **1e** (EML979), and **1f** (EML980) on PRMT9 activity in MCF7 (top) and MDA-MB-436 breast cancer cell lines. MCF7 and MDA-MB-436 cells were treated with 4 candidate inhibitors at indicated concentrations for 72 h. The total cell lysates were harvested in RIPA buffer and the levels of SF3B2 R508me2s, SF3B2, and PRMT9 were detected by using Western blot assays. Anti-Tubulin antibody was used as a loading control.

However, as shown in Figure 8, in both cell lines and for all of the tested compounds, we were not able to see a convincing inhibition on the levels of SF3B2 R508 methylation. As mentioned above, this is not surprising considering the low cell permeability of these compounds.

### Quantification of in Cell Methylation by Mass Spectrometry

Antibody-based methods such as enzyme-linked immunosorbent assay (ELISA) or Western blot are widely used to detect PTMs, yet they are significantly less sensitive than methods based on mass spectrometry, in which resolution is based on mass changes and includes a variety of PTMs within a certain mass range in a single measurement. This is even more evident in the case of the combination of state-of-the-art spectrometers with high resolving power and powerful bioinformatic tools, that made very popular the use of “label-free” quantification methods (LFQ) as an alternative to stable isotope labeling strategies.^68,69^ Therefore, we decided to investigate whether variations in the level of arginine methylation of specific substrates of PRMT7 and PRMT9 could be detected by LFQ mass spectrometry. To this aim, we focused on the heat shock 70 kDa protein 1B (HSP70) and the heterogeneous nuclear ribonucleoprotein A1 (HNRNPA1), which are methylated by PRMT7 on R469 and R194, respectively,^9,70^ and on SF3B2, symmetrically dimethylated on R508 by PRMT9.^11,13^

**Figure 9:**
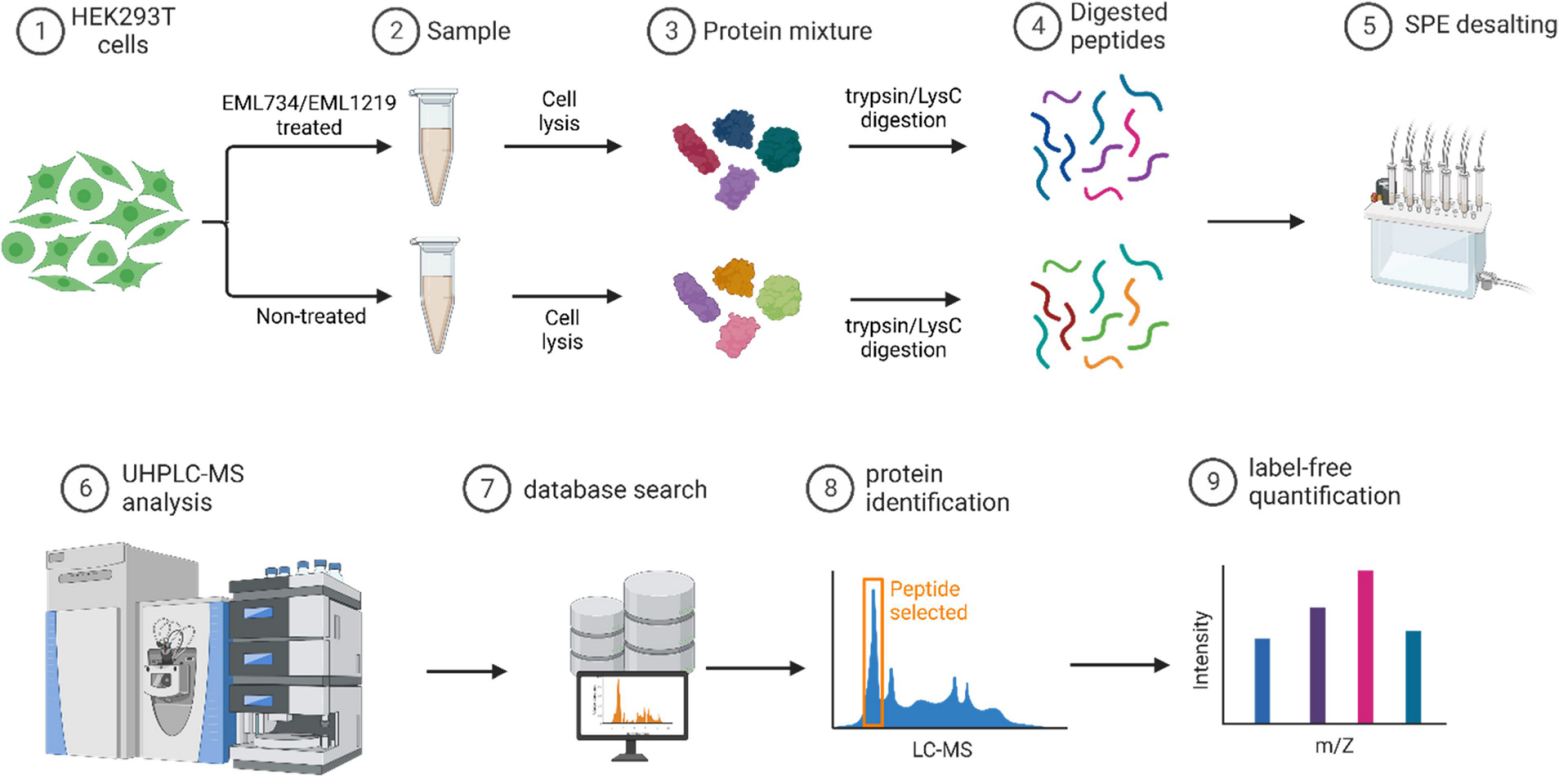
Schematic description of the MS proteomic experiment. Created with BioRender.com.

Briefly, HEK293T cells treated with compounds **1a** or **1j** or untreated were lysed through sonication, and then proteins from each lysate were denatured and digested with a protease (trypsin/LysC) into a peptide mixture, which was subsequently analyzed by tandem MS (MS/MS), identified by database searching, and quantified (Figure 9; see Experimental Section for details).

**Figure 10:**
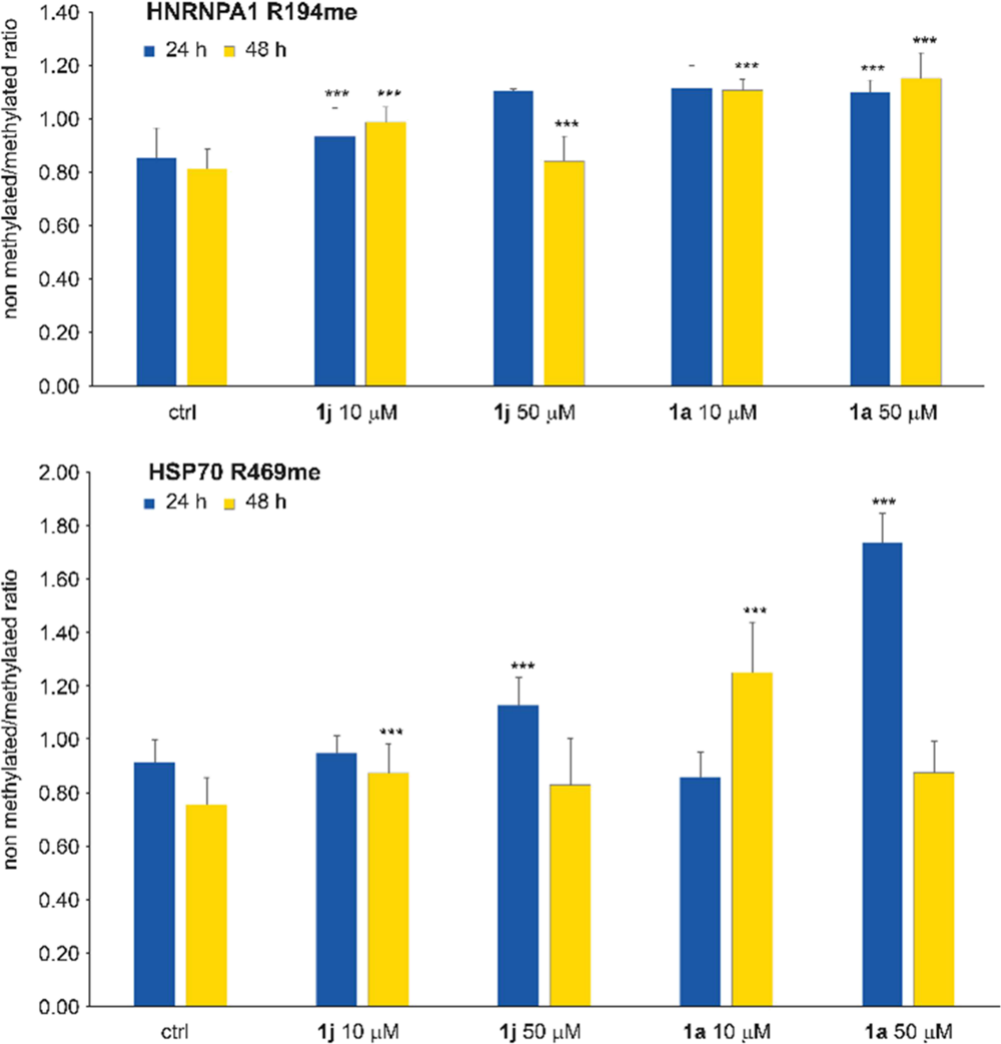
Compounds **1a** and **1j** inhibit PRMT7 in cells. The bar graphs plot the ratio between the abundance of nonmethylated over methylated peptides in HEK392T cells treated with compounds **1a** or **1j** or untreated for 24 h (blue) or 48 h (yellow). The top panel shows the ratio between HNRNPA1 unmethylated over R194me peptides, and the bottom panel shows the ratio between HSP70 unmethylated over R469me peptides.

For both untreated and compound-treated samples, the ratio between the abundance of the nonmethylated over the monomethylated peptides was calculated and plotted as bar graphs. As shown in Figure 10, for both substrates of PRMT7, HNRNPA1, and HSP70, the ratio increased in treated samples compared to control, indicating an increasing prevalence of unmethylated over methylated peptides, thus revealing a slight but significant inhibition of the enzyme catalytic activity even in cells. The effect is concentration- and time-dependent.

Unfortunately, after trypsin/Lys-C digestion, we were not able to identify the SF3B2 peptide(s) including R508 but only peptides preceding or following it (e.g., 496–507 or 515–530). Therefore, we resolved to repeat the experiments using digestion by the alternative protease Glu-C or a digestion with trypsin/Lys-C and Glu-C in parallel and subsequent MS-proteomics analysis.^71^ Alas, both attempts were not successful, and again we identified the protein but not the peptide(s) including R508. Nonetheless, the results obtained for two distinct substrates of PRMT7 confirmed that, although nonoptimized for cell permeability, compounds **1a** and **1j** inhibit PRMT activity also in a cellular context.

## Conclusions

Compared to other PRMTs, PRMT7 and PRMT9 are relatively underinvestigated and, although they have been recently identified as potential therapeutic targets for the treatment of various diseases, including different types of cancer,^18,19,22−24,25^ much is still to be understood on their biological roles, as well as on the structural requirements that could drive the development of selective modulators of their methyltransferase activity.

Prompted by our longstanding interest in PRMTs, we recently demonstrated that modulating the distance between pharmacophoric moieties of compounds spanning both the substrate and the cosubstrate pockets leads to potent and selective PRMT4 inhibitors.^31^ In this work, starting from the reanalysis of those data, we observed that, differently from PRMT4, PRMT7 seems to preferentially bind derivatives with shorter linkers, consistently with its previously described restrictive and narrow active site.^15,47^ The shortest compound in the series (**1a**, EML 734), featuring a propyl spacer between the 4-hydroxy-2-naphthoate moiety and the guanidine group, showed an IC_50_ value of 0.32 μM and a certain selectivity compared to other tested PRMTs (SI values in the range 26–227). The trend was confirmed by the lower homologue **1i** (EML1102), in which the reduction of the spacer length (propyl to ethyl) further reduced the inhibitory potency against PRMT1, PRMT3, PRMT6, and PRMT8 but substantially maintained the inhibitory activity of **1a** against PRMT7.

This prompted us to extend the study to PRMT9, and we decided to gauge the inhibitory activity of compounds **1a**–**i**. As a primary screening assay, we used an in-house custom-developed AlphaLISA assay employing a biotinylated 20-amino acid peptide of SF3B2 (aa 500–519) as a substrate. We found that compound **1a** is also a very good inhibitor of PRMT9 with an IC_50_ value in the submicromolar range (IC_50_ = 0.89 μM) and a good selectivity profile against the other PRMTs. In the case of PRMT9, the distance between the methyl 4-hydroxy-2-naphthoate moiety and the arginine-mimetic group does not significantly affect the inhibitory activity of the compounds, with all the compounds **1a**–**e** (*n* = 1–5) showing comparably good inhibiting properties (IC_50_ values around 1 μM) against this enzyme. Differently from what we observed for PRMT7, the further reduction of the length of the alkyl spacer between the 4-hydroxy-2-naphthoate moiety and the guanidine group as featured by compound **1i** was detrimental for the inhibition of PRMT9, with a 3-fold reduction in potency. Similarly, a decrease of the inhibitory activity was observed when the linker between the guanidine group and the adenosine moiety was more than a two-carbon atom long, with compounds **1g** and **1h** being the least effective PRMT9 inhibitors in the series.

A radioisotope-based assay was used as a secondary screening approach and confirmed the PRMT9-inhibiting activity of the compounds. Docking calculations with the crystal structures of PRMT9 and PRMT7 proposed binding modes for **1a** that were confirmed by 500 ns long molecular dynamics simulations. In the interaction with PRMT9, the ligand adenine ring is able to occupy the SAM cofactor binding pocket, establishing H-bond interactions with the L208 and S236 backbone NHs groups.

Additional H-bonds are formed by the sugar OH groups with the E255 backbone CO, while the arginine-mimetic group is involved in ionic contacts with the negatively charged side chains of the double E loop (E150 and E264). E264 is also involved in charged-reinforced H-bonds with the urea moiety. Finally, the 4-hydroxy-2-naphthoate group is inserted in a rather lipophilic cleft establishing a π–π interaction with the W152 side chain, while the methyl ester in position 6 is H-bonding E433 backbone NH. Also in the binding with PRMT7, the ligand adenosine and sugar rings are involved in H-bond interactions reminiscent of the interactions established by the same rings in the SAM cosubstrate. Additionally, the guanidine portion is involved in ionic contacts with negatively charged residues. The main differences, however, are recorded for the terminal 4-hydroxy-2-naphthoate group that in this case is pointing toward a rather solvent-exposed and hydrophilic protein region.

To further validate the ligand/PRMT9 interaction model, we designed and synthesized a trifluoromethylated analogue of **1a** (namely, **1j**, EML1219) with the aim to strengthen the π–π contact with the W152 side chain of the enzyme without altering the overall conformation. SPR studies confirmed a specific and strong binding interaction between PRMT9 and **1j** with a *K*_D_ value in the submicromolar range and a relatively high in vitro residence time value (*K*_D_ = 188 nM; τ_R_ = 500 s).

However, this gain in affinity was paid by selectivity against the other PRMTs, particularly against the other type II enzyme PRMT5, as well as against related methyltransferases like the non-SET domain containing DOT1L. On the contrary, **1j** was found to be selective against a panel of SET-domain-containing proteins including ASH1L/KMT2H, EZH2/KMT6, MLL1/KMT2A, SET7/9/KMT7, SETD8/KMT5A, SUV39H2/KMT1B, and SUV420H1/KMT5B, which were not inhibited even at the higher tested concentration (100 μM, > 500 fold higher than the IC_50_ value against PRMT9).

Similar to what we previously observed for compound **1h**, LFQ mass spectrometry revealed that compounds **1a** and **1j** are able to affect PRMT activity even in a cellular context, regardless of their low cell permeability.

In conclusion, this study sheds more light on the binding interactions with PRMT7 and PRMT9 of inhibitors spanning both the substrate and the cosubstrate pockets and provides structural information that could inform the development of potent and selective inhibitors of these two enzymes.

### Chemistry

**Scheme 1:**
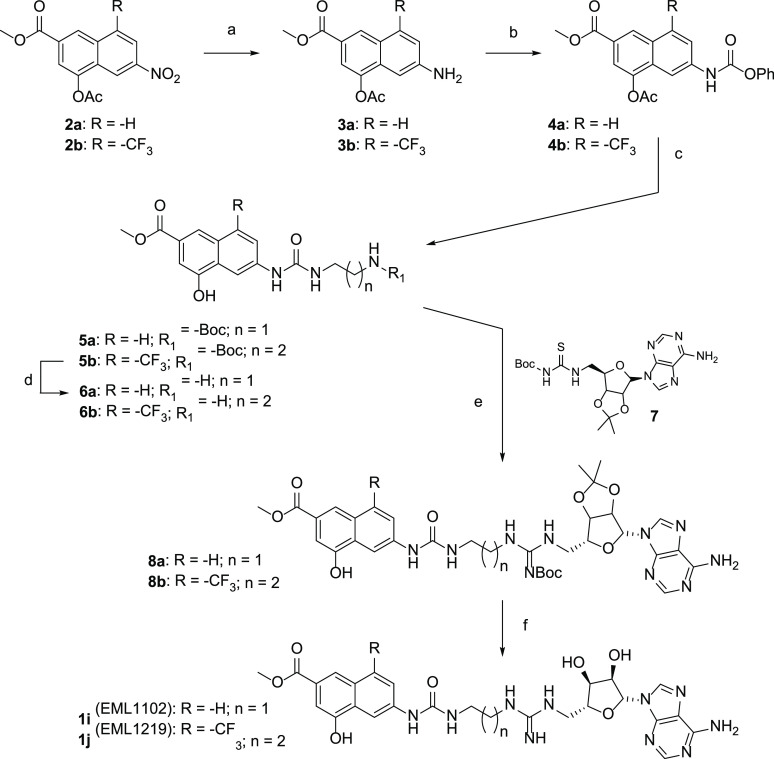
Synthesis of Compounds **1i** and **1j** Reagents and conditions: (a) zinc dust, acetic acid, 1 h (97–98%); (b) phenyl chloroformate, TEA, AcOEt, r. t., 12 h (65–70%); (c) TEA, dry DMF, r. t., 2 h (68–70%); (d) DCM/TFA 9:1, r. t., 2 h (80–92%); (e) EDC hydrochloride, TEA, dry DCM, r. t., 18 h (60–74%); (f) DCM/TFA 1:1, r. t., 2 h (60–76%).

The synthetic protocol adopted for the preparation of compounds **1i** and **1j** is depicted in Scheme 1.

**Scheme 2:**
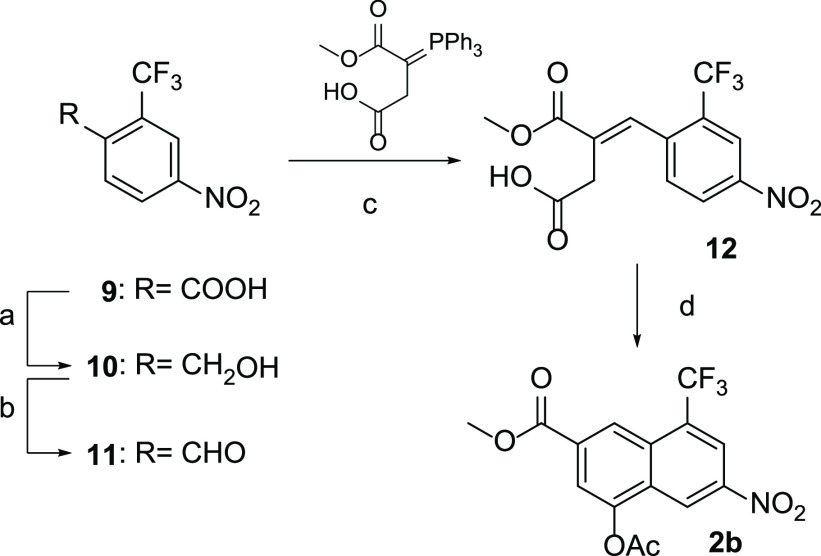
Synthesis of Derivative **2b** Reagents and conditions: (a) NaBH_4_, I_2_, dry THF, 0 °C to reflux, 18 h (88%); (b) Dess-Martin periodinane, dry DCM, 2 h, r.t. (74%); (c) toluene, r. t., 48 h (63%); (d) sodium acetate, acetic anhydride, 120 °C (MW), 25 min (67%).

4-acetoxy-6-nitro-2-naphthoate (**2a**) and 4-acetoxy-6-nitro-8-(trifluoromethyl)-2-naphthoate (**2b**) were prepared according to the synthetic procedures previously reported by us (Scheme 2).^31^ Reduction of the nitro group with zinc dust in acetic acid (**3a, 3b**), followed by treatment with phenyl chloroformate, allowed us to obtain compounds **4a, 4b**, which straightforwardly reacted with the proper mono-Boc-protected alkyldiamines to yield ureidic compounds **5a, 5b**. After trifluoroacetic acid (TFA) deprotection, the corresponding amines **6a, 6b** were coupled with the adenosine derivative **7**(31) in the presence of EDC hydrochloride as an activating agent. The obtained derivatives **8a,8b** were finally subjected to acidic deprotection to give the desired compounds **1i** and **1j**.

## Experimental Section

### Chemistry

#### General Directions

All chemicals purchased from Merck KGaA and Fluorochem Ltd. were of the highest purity. All solvents were reagent grade and, when necessary, were purified and dried by standard methods. All reactions requiring anhydrous conditions were conducted under a positive atmosphere of nitrogen in oven-dried glassware. Standard syringe techniques were used for the anhydrous addition of liquids. Reactions were routinely monitored by TLC performed on aluminum-backed silica gel plates (Merck KGaA, Alufolien Kieselgel 60 F254) with spots visualized by UV light (λ = 254, 365 nm) or using a KMnO4 alkaline solution. Solvents were removed by using a rotary evaporator operating at a reduced pressure of ∼10 Torr. Organic solutions were dried over anhydrous Na_2_SO_4_. Chromatographic purification was done on an automated flash-chromatography system (Isolera Dalton 2000, Biotage) using cartridges packed with KPSIL, 60 Å (40–63 μm particle size). All microwave-assisted reactions were conducted in a CEM Discover SP microwave synthesizer equipped with a vertically focused IR temperature sensor. Analytical high-performance liquid chromatography (HPLC) was performed on a Shimadzu SPD 20A UV/vis detector (λ = 220 and 254 nm) using a C-18 column Phenomenex Synergi Fusion-RP 80A (75 × 4.60 mm; 4 μm) at 25 °C using mobile phases A (water + 0.1% TFA) and B (ACN + 0.1% TFA) at a flow rate of 1 mL/min. Preparative HPLC was performed using a Shimadzu Prominence LC-20AP instrument with the UV detector set to 220 and 254 nm. Samples were injected into a Phenomenex Synergi Fusion-RP 80A (150 × 21 mm; 4 mm) C-18 column at room temperature. Mobile phases A (water + 0.05% TFA) and B (ACN + 0.03% TFA) were used at a flow rate of 20 mL/min. ^1^H and ^19^F spectra were recorded at 400 MHz on a Bruker Ascend 400 spectrometer, while ^13^C NMR spectra were obtained by distortionless enhancement by polarization transfer quaternary (DEPTQ) spectroscopy on the same spectrometer. Chemical shifts are reported in δ (ppm) relative to the internal reference tetramethylsilane (TMS). For ^19^F spectra, trifluorotoluene (−62.74 ppm) was used as an external standard. Low-resolution and high-resolution mass spectra were recorded on a ThermoFisher Scientific Orbitrap XL mass spectrometer in electrospray positive ionization modes (ESI-MS). All tested compounds possessed a purity of at least 95% established by HPLC unless otherwise noted.

### Methyl 6-(3-(2-(3-(((2*R*,3*S*,4*R*,5*R*)-5-(6-Amino-9*H*-purin-9-yl)-3,4-dihydroxytetrahydrofuran-2-yl)methyl)guanidino)ethyl)ureido)-4-hydroxy-2-naphthoate (**1i**)

Compound **8a** (0.100 g, 0.120 mmol) was dissolved in a 1:1 DCM/TFA solution (0.1 M), and then a drop of water was added. The resulting mixture was stirred at room temperature for 2 h. Then, the solvent was evaporated and the crude material was purified by reversed-phase high-performance liquid chromatography (RP-HPLC) to afford the TFA salt of **1i** as a white solid (54.0 mg, 76%). ^1^H NMR (400 MHz, DMSO-*d*_*6*_) δ 10.34 (s, 1H, exchangeable with D_2_O), 9.17 (s, 1H, exchangeable with D_2_O), 8.52 (s, 1H), 8.34 (s, 1H), 8.27–8.21 (m, 1H), 7.97 (s, 1H), 7.89 (d, *J* = 9.0 Hz, 1H), 7.63–7.38 (m, 6H, 4H, exchangeable with D_2_O), 7.30 (s, 1H), 6.49 (br t, *J* = 5.7 Hz, 1H, exchangeable with D_2_O), 5.95 (d, *J* = 5.7 Hz, 1H), 4.69 (t, *J* = 5.5 Hz, 1H), 4.18–4.12 (m, 1H), 4.09–4.00 (m, 1H), 3m.86 (s, 3H), 3.28–3.17 (m, 4H). ^13^C NMR (100 MHz, DMSO-*d*_*6*_) δ: 167.1, 156.6, 156.0, 153.1, 150.3, 149.4, 141.6, 140.0, 130.3, 129.4, 128.3, 125.3, 121.7, 120.7, 119.6, 115.1, 108.0, 107.0, 88.3, 82.8, 73.3, 71.6, 52.5, 43.7, 41.9, 38.8. HRMS (ESI): *m*/*z* [M + H]^+^ calcd for C_26_H_30_N_10_O_7_ + H^+^: 595.2372. Found: 595.2376.

### Methyl 6-(3-(3-(3-(((2*R*,3*S*,4*R*,5*R*)-5-(6-Amino-9*H*-purin-9-yl)-3,4-dihydroxytetrahydrofuran-2-yl)methyl)guanidino)propyl)ureido)-4-hydroxy-8-(trifluoromethyl)-2-naphthoate (**1j**)

The TFA salt of compound **1j** was obtained as a white solid (22.0 mg, 60%), starting from compound **8b** (38.0 mg, 0.046 mmol), following the procedure described for **1i**. ^1^H NMR (400 MHz, DMSO-*d*_*6*_) δ 10.80 (s, 1H, exchangeable with D_2_O), 9.36 (s, 1H, exchangeable with D_2_O), 8.47 (d, *J* = 2.2 Hz, 1H), 8.43 (s, 1H), 8.26 (d, *J* = 2.3 Hz, 1H), 8.25 (s, 1H), 8.09 (s, 1H), 7.82 (s, 2H, exchangeable with D_2_O), 7.51–7.45 (m, 1H, exchangeable with D_2_O), 7.45–7.39 (m, 3H, 2H, exchangeable with D_2_O), 6.55 (br t, *J* = 5.7 Hz, 1H, exchangeable with D_2_O), 5.93 (d, *J* = 5.8 Hz, 1H), 4.71 (t, *J* = 5.4 Hz, 1H), 4.16 (t, *J* = 4.5 Hz, 1H), 4.05–4.00 (m, 1H), 3.89 (s, 3H), 3.19–3.10 (m, 4H), 1.69–1.61 (m, 2H). ^19^F NMR (377 MHz, DMSO-*d*_*6*_) δ: −58.57 (s, 3F), −73.97 (s, 3F). ^13^C NMR (101 MHz, DMSO-*d*_*6*_) δ: 166.5, 158.8, 158.5, 156.2, 155.5, 154.4, 153.5, 150.6, 149.2, 141.2, 138.6, 128.9, 127.0, 123.8, 119.8, 119.4, 116.3, 111.9, 107.6, 88.2, 82.7, 73.0, 71.2, 52.7, 43.4, 36.8, 29.4. HRMS (ESI): *m*/*z* [M + H]^+^ calcd for C_28_H_31_F_3_N_10_O_7_ + H^+^: 677.2402. Found: 677.2401.

### Methyl 4-Acetoxy-6-nitro-8-(trifluoromethyl)-2-naphthoate (**2b**)

A 10 mL CEM pressure vessel equipped with a stir bar was charged with **12** (0.350 g, 1.05 mmol), acetic anhydride (2.5 mL), and sodium acetate (0.129 g, 1.58 mmol). The microwave vial was sealed and heated in a CEM Discover microwave synthesizer to 120 °C (measured by the vertically focused IR temperature sensor) for 25 min. After cooling to room temperature, the reaction mixture was filtered, and the filtrate was concentrated under reduced pressure. The title product was obtained as a yellow solid (0.250 g, 67%) after recrystallization from AcOEt. ^1^H NMR (400 MHz, DMSO-*d*_*6*_) δ 9.17 (d, *J* = 1.4 Hz, 1H), 8.70 (d, *J* = 2.2 Hz, 1H), 8.67 (s, 1H), 8.22 (d, *J* = 1.4 Hz, 1H), 3.99 (s, 3H), 2.55 (s, 3H). MS (ESI) *m*/*z*: 358 (M + H)^+^.

### Methyl 4-Acetoxy-6-amino-8-(trifluoromethyl)-2-naphthoate (**3b**)

To a solution of **2b** (0.150 g, 0.420 mmol) in acetic acid (9 mL) was added Zn dust (0.275 g, 4.20 mmol). The resulting mixture was stirred for 1 h at room temperature, filtered, and concentrated in vacuo. The acid residue was dissolved in a saturated aqueous solution of NaHCO_3_ (30 mL) and extracted with AcOEt (3 × 30 mL). The collected organic phases were washed with brine (30 mL), dried over Na_2_SO_4_, filtered, and concentrated under reduced pressure. The title compound **3b** (0.134 g, 97%) was obtained as a pale-yellow solid, which was used in the next step without further purification. ^1^H NMR (400 MHz, DMSO-*d*_6_) δ 8.39 (s, 1H), 7.69 (d, *J* = 1.4 Hz, 1H), 7.59 (d, *J* = 2.2 Hz, 1H), 7.09–7.04 (m, 1H), 6.39 (s, 2H, exchangeable with D_2_O), 3.89 (s, 3H), 2.44 (s, 3H). MS (ESI) *m*/*z*: 328 (M + H)^+^.

### Methyl 4-Acetoxy-6-((phenoxycarbonyl)amino)-8-(trifluoromethyl)-2-naphthoate (**4b**)

To a solution of **3b** (0.134 g, 0.409 mmol) in 1.7 mL of AcOEt was added TEA (0.063 mL, 0.45 mmol). The resulting mixture was cooled at 0 °C, and phenyl chloroformate (0.057 mL, 0.45 mmol) was added dropwise. The resulting yellow suspension was allowed to warm at room temperature and stirred for 16 h. Then, the reaction mixture was diluted with AcOEt (30 mL) and washed with water (3 × 20 mL), HCl 1N (3 × 20 mL), saturated aqueous solution of NaHCO_3_ (3 × 20 mL), and brine (30 mL). The organic phase was dried in Na_2_SO_4_, filtered, and concentrated under reduced pressure. The title compound was obtained as a pale-yellow solid (0.122 g, 65%) after recrystallization from AcOEt. ^1^H NMR (400 MHz, DMSO-*d*_6_) δ 10.99 (s, 1H, exchangeable with D_2_O), 8.55 (s, 1H), 8.43 (s, 1H), 8.37–8.32 (m, 1H), 7.94 (d, *J* = 1.4 Hz, 1H), 7.51–7.41 (m, 2H), 7.35–7.26 (m, 3H), 3.94 (s, 3H), 2.44 (s, 3H). MS (ESI) *m*/*z*: 448 (M + H)^+^.

### Methyl 6-(3-(2-((*tert*-Butoxycarbonyl)amino)ethyl)ureido)-4-hydroxy-2-naphthoate (**5a**)

To a stirring solution of compound **4a** (0.400 g, 1.05 mmol) in dry DMF (5 mL) were added a solution of *tert*-butyl (2-aminoethyl)carbamate (0.336 g, 2.10 mmol) and TEA (0.294 mL, 2.10 mmol) in dry DMF (5 mL). The resulting reaction mixture was stirred at room temperature for 2 h. Then, a saturated aqueous solution of NaHCO_3_ was added (50 mL) and the resulting mixture was extracted with AcOEt (3 × 30 mL). The combined organic phases were washed with a saturated aqueous solution of NaHCO_3_ (3 × 20 mL) and brine (10 mL), dried over Na_2_SO_4_, filtered, and concentrated under reduced pressure. The crude material was purified by flash chromatography to afford the title compound as an orange solid (0.290 g, 68%). ^1^H NMR (400 MHz, DMSO-d_6_) δ 10.28 (s, 1H, exchangeable with D_2_O), 8.91 (s, 1H, exchangeable with D_2_O), 8.27 (d, *J* = 2.1 Hz, 1H), 7.97 (s, 1H), 7.89 (d, *J* = 8.9 Hz, 1H), 7.55 (dd, *J* = 8.9, 2.1 Hz, 1H), 7.30 (s, 1H), 6.85 (t, *J* = 5.0 Hz, 1H, exchangeable with D_2_O), 6.25 (t, *J* = 5.7 Hz, 1H, exchangeable with D_2_O), 3.87 (s, 3H), 3.14–3.08 (m, 2H), 3.01–2.94 (m, 2H), 1.40 (s, 9H). MS (ESI) *m*/*z*: 404 (M + H)^+^.

### Methyl 6-(3-(3-((*tert*-Butoxycarbonyl)amino)propyl)ureido)-4-hydroxy-8-(trifluoromethyl)-2-naphthoate (**5b**)

Compound **5b** was obtained as a pale-yellow solid (67.0 mg, 70%), starting from compound **4b** (88.0 mg, 0.197 mmol) and *tert*-butyl (3-aminopropyl)carbamate (68.0 mg, 0.390 mmol), following the procedure described for **5a**. ^1^H NMR (400 MHz, DMSO-*d*_6_) δ 10.76 (s, 1H, exchangeable with D_2_O), 9.27 (s, 1H, exchangeable with D_2_O), 8.47 (d, *J* = 2.2 Hz, 1H), 8.24 (d, *J* = 2.2 Hz, 1H), 8.09 (s, 1H), 7.42 (d, *J* = 1.4 Hz, 1H), 6.83 (t, *J* = 5.7 Hz, 1H, exchangeable with D_2_O), 6.34 (t, *J* = 5.8 Hz, 1H, exchangeable with D_2_O), 3.89 (s, 3H), 3.15–3.08 (m, 2H), 3.01–2.94 (m, 2H), 1.60–1.52 (m, 2H), 1.38 (s, 9H). MS (ESI) *m*/*z*: 486 (M + H)^+^.

### Methyl 6-(3-(2-Aminoethyl)ureido)-4-hydroxy-2-naphthoate (**6a**)

Compound **5a** (0.700 g, 1.73 mmol) was dissolved in 10 mL of a solution of DCM/TFA (9:1), and the mixture was stirred at room temperature for 2 h. Then, the solvent was evaporated, and the resulting solid was washed with CHCl_3_ to give the TFA salt of compound **6a** as a white solid (0.558 g, 80%). ^1^H NMR (400 MHz, DMSO-*d*_*6*_) δ 10.33 (s, 1H, exchangeable with D_2_O), 9.19 (s, 1H, exchangeable with D_2_O), 8.29 (d, *J* = 2.2 Hz, 1H), 7.98–7.95 (m, 1H), 7.90 (d, *J* = 8.9 Hz, 1H), 7.76 (br s, 3H, exchangeable with D_2_O), 7.58 (dd, *J* = 8.9, 2.1 Hz, 1H), 7.30 (s, 1H), 6.47 (br t, 1H, *J* = 5.8 Hz, exchangeable with D_2_O), 3.86 (s, 3H), 3.29–3.12 (m, 2H), 2.98–2.89 (m, 2H). MS (ESI) *m*/*z*: 303 (M + H)^+^.

### Methyl 6-(3-(3-Aminopropyl)ureido)-4-hydroxy-8-(trifluoromethyl)-2-naphthoate (**6b**)

The TFA salt of compound **6b** was obtained as a white solid (63.4 mg, 92%), starting from compound **5b** (67.0 mg, 0.138 mmol), following the procedure described for **6a**. ^1^H NMR (400 MHz, DMSO-*d*_*6*_) δ 10.80 (s, 1H, exchangeable with D_2_O), 9.42 (s, 1H, exchangeable with D_2_O), 8.49 (d, *J* = 2.2 Hz, 1H), 8.27 (d, *J* = 2.2 Hz, 1H), 8.10 (s, 1H), 7.76–7.63 (m, 3H, exchangeable with D_2_O), 7.43 (d, *J* = 1.4 Hz, 1H), 6.65 (t, *J* = 5.9 Hz, 1H, exchangeable with D_2_O), 3.89 (s, 3H), 3.24–3.17 (m, 2H), 2.88–2.80 (m, 2H), 1.79–1.72 (m, 2H). MS (ESI) *m*/*z*: 386 (M + H)^+^.

### Methyl 6-(3-(2-((*E*)-3-(((3*aR*,4*R*,6*R*,6*aR*)-6-(6-Amino-9*H*-purin-9-yl)-2,2-dimethyltetrahydrofuro[3,4-*d*][1,3]dioxol-4-yl)methyl)-2-(*tert*-butoxycarbonyl)guanidino)ethyl)ureido)-4-hydroxy-2-naphthoate (**8a**)

To a stirred suspension of **6a** (0.147 g, 0.354 mmol) and **7** (100 mg, 0.177 mmol) in dry DCM, EDC hydrochloride (69.0 mg, 0.354 mmol) and TEA (0.074 mL, 0.531 mmol) were added, and the resulting mixture was stirred at room temperature for 18 h. Then, the solvent was evaporated under reduced pressure, and the resulting oil was taken up with water. The aqueous phase was extracted with AcOEt (3 × 25 mL), and the collected organic phases were washed with brine, dried over Na_2_SO_4_, filtered, and concentrated under reduced pressure. The crude material was purified by flash chromatography, yielding **8a** as a white solid (0.109 g, 74%). ^1^H NMR (400 MHz, DMSO-*d*_*6*_) δ 10.28 (s, 1H, exchangeable with D_2_O), 9.01–8.90 (m, 1H, exchangeable with D_2_O), 8.35 (s, 1H), 8.23 (d, *J* = 2.2 Hz, 1H), 8.18 (s, 1H), 7.96 (d, *J* = 1.4 Hz, 1H), 7.88 (d, *J* = 8.9, 1H), 7.59 (dd, *J* = 8.9, 2.2 Hz, 1H), 7.33 (s, 2H, exchangeable with D_2_O), 7.29 (d, *J* = 1.4 Hz, 1H), 6.42–6.32 (m, 1H, exchangeable with D_2_O), 6.15 (s, 1H), 5.47–5.39 (m, 1H), 5.10–4.93 (m, 1H), 4.35–4.24 (m, 1H), 3.86 (s, 3H), 3.52–3.40 (m, 2H), 3.28–3.22 (m, 4H), 1.52 (s, 3H), 1.36 (s, 9H), 1.31 (s, 3H); MS (ESI) *m*/*z*: 835 (M + H)^+^.

### Methyl 6-(3-(3-((*E*)-3-(((3*aR*,4*R*,6*R*,6*aR*)-6-(6-Amino-9*H*-purin-9-yl)-2,2-dimethyltetrahydrofuro[3,4-*d*][1,3]dioxol-4-yl)methyl)-2-(*tert*-butoxycarbonyl)guanidino)propyl)ureido)-4-hydroxy-8-(trifluoromethyl)-2-naphthoate (**8b**)

The compound **8b** was obtained as a white solid (42.0 mg, 60%), starting from compound **6b** (63.4 mg, 0.127 mmol) and compound **7** (39.3 mg, 0.085 mmol), following the procedure described for **8a**. ^1^H NMR (400 MHz, DMSO-*d*_*6*_) δ 10.76 (s, 1H, exchangeable with D_2_O), 9.21 (s, 1H, exchangeable with D_2_O), 8.47 (d, *J* = 2.4 Hz, 1H), 8.35 (d, *J* = 2.2 Hz, 1H), 8.25 (d, *J* = 2.4 Hz, 1H), 8.18 (d, *J* = 2.2 Hz, 1H), 8.09 (s, 1H), 7.42 (d, *J* = 1.4 Hz, 1H), 7.34 (s, 2H, exchangeable with D_2_O), 6.50–6.42 (m, 1H, exchangeable with D_2_O), 6.15 (s, 1H,), 5.78–5.73 (m, 1H), 5.07–4.97 (m, 1H), 4.33–4.25 (m, 1H), 3.89 (s, 3H), 3.49–3.41 (m, 2H), 3.22–3.11 (m, 4H), 1.68–1.60 (m, 2H), 1.53 (s, 3H), 1.35 (s, 9H), 1.32 (s, 3H). MS (ESI) *m*/*z*: 817 (M + H)^+^.

### (4-Nitro-2-(trifluoromethyl)phenyl)methanol (**10**)

To a cooled solution of 4-nitro-2-(trifluoromethyl)benzoic acid (**9**; 3.00 g, 12.76 mmol) in dry THF (26 mL) was added NaBH_4_ (1.21 g, 31.9 mmol) portion-wise. Subsequently, a solution of I_2_ (3.24 g, 12.76 mmol) in 13 mL of dry THF was added over 1 h, and the resulting mixture was stirred at room temperature for 1 h and then refluxed for 12 h. The mixture was cooled at room temperature, and a solution of KOH 20% (100 mL) was added and stirred for 1 h: the aqueous phase was extracted with AcOEt (3 × 40 mL), and the collected organic phases were washed with brine, dried over Na_2_SO_4_, filtered, and concentrated under reduced pressure. The crude product was purified by flash chromatography, yielding **10** as a yellow solid (2.50 g, 88%). ^1^H NMR (400 MHz, DMSO-*d*_*6*_) δ 8.55 (dd, *J* = 8.6, 2.4 Hz, 1H), 8.39 (d, *J* = 2.4 Hz, 1H), 8.08 (d, *J* = 8.6 Hz, 1H), 5.95–5.88 (m, 1H, exchangeable with D_2_O), 4.78 (s, 2H). MS (ESI) *m*/*z*: 222 (M + H)^+^.

### 4-Nitro-2-(trifluoromethyl)benzaldehyde (**11**)

To a cooled solution of **10** (1.3 g, 5.88 mmol) in dry DCM (25 mL) was added Dess-Martin periodinane (2.47 g, 6.47 mmol) portion-wise, and the resulting mixture was stirred at room temperature for 3 h. The formed-white precipitate was filtered off, and the filtrate was taken up with DCM (60 mL). The organic phase was washed with saturated aqueous solution of NaHCO_3_ (3 × 30 mL) and brine (30 mL), dried over Na_2_SO_4_, filtered, and concentrated under reduced pressure. The crude was purified by flash chromatography, yielding **11** as a pale-yellow solid (0.950 g, 74%). ^1^H NMR (400 MHz, DMSO-*d*_*6*_) δ 10.34 (s, 1H), 8.69 (dd, *J* = 8.5, 2.2 Hz, 1H), 8.57 (d, *J* = 2.2 Hz, 1H), 8.33 (d, *J* = 8.5 Hz, 1H). MS (ESI) *m*/*z*: 220 (M + H)^+^.

### (*E*)-3-(Methoxycarbonyl)-4-(4-nitro-2-(trifluoromethyl)phenyl)but-3-enoic acid (**12**)

To a suspension of 4-methoxy-4-oxo-3-(triphenyl-l5-phosphaneylidene)butanoic acid (1.13 g, 2.88 mmol) in toluene (20 mL) was added compound **11** (0.630 g, 2.88 mmol). The resulting mixture was stirred at room temperature for 48 h and then concentrated under reduced pressure. The residue was taken up with saturated aqueous solution of NaHCO_3_ (60 mL), washed with Et_2_O (3 × 30 mL), and acidified with HCl 6 N until pH 2. The aqueous phase was extracted with AcOEt (3 × 30 mL), and the collected organic phases were washed with brine, dried over Na_2_SO_4_, filtered, and concentrated in vacuo to afford **12** (0.600 g, 63%) as a yellow solid. ^1^H NMR (400 MHz, DMSO-*d*_*6*_) δ 8.59 (dd, *J* = 8.5, 2.4 Hz, 1H), 8.50 (d, *J* = 2.4 Hz, 1H), 7.86 (d, *J* = 2.4 Hz, 1H), 7.72 (d, *J* = 8.5 Hz, 1H), 3.79 (s, 3H), 3.23 (s, 2H); MS (ESI) *m*/*z*: 334 (M+H)^+^.

### AlphaLISA PRMT9 Activity Assay

PRMT9 activity assays were performed by AlphaLISA using the “PRMT9 Homogeneous assay Kit” (BPS BioScience, #52069), as opportunely modified by us (see above in the text).

The assays were performed in white opaque OptiPlate-384 (PerkinElmer, no. 6007299) at 22 °C in a final volume of 30 μL, using the HMT assay buffer 2A (BPS-BioScience #52170-A).

In each well, 2 μL of human recombinant PRMT9 (BPS BioScience, no. 79124) (50 ng/μL) was first incubated for 30 min with 3 μL of each compound (dissolved in DMSO and diluted in assay buffer to obtain 1% DMSO). Then, each well was added with 0.5 μL of the biotinylated substrate peptide SF3B2 (aa 500–519) (Pepmic, custom synthesis) (final concentration, 100 nM), 1 μL of SAM 250 μM, 1.5 μL of water, and 2 μL of 4× HMT assay buffer 2A to reach the final volume of 10 μL. The reaction was incubated for 60 min. Afterward, in each well, 5 μL of a 1:100 dilution of Primary antibody 28 (BPS BioScience, #52140Z3) in Detection buffer (BPS BioScience) and 5 μL of anti-Rabbit acceptor beads (PerkinElmer, #AL104C) were added to obtain a final concentration of 20 μg/mL. After an incubation of 60 min, 10 μL of streptavidin donor beads (PerkinElmer, # 6760002) diluted in detection buffer was added in each well (final concentration, 20 μg/mL). After an incubation of 30 min, signals were read in Alpha mode with a PerkinElmer EnSight multimode microplate reader (excitation at 680 nm and emission at 615 nm).

For each incubation step, the OptiPlate was sealed with a protective foil to prevent evaporation and contamination. Donor and Acceptor beads were added to the mixture in subdued light.

The 100% activity (positive control) was reached using vehicle (DMSO), while 0% activity (negative control) was obtained without the protein. Data were analyzed by using Excel and Prism software. Values obtained for each compound are mean ± SD determined for three separate experiments.

### PRMT7 and PRMT9 Radioisotope-Based Activity Assay

#### Protein Purification and Inhibitors

Human PRMT9, *C. elegans* PRMT9, human PRMT7, and *C. elegans* PRMT7 plasmids were sequenced, expressed in *E. coli* as GST-fusion proteins, and purified as previously described.^48^ Substrates were also sequenced and purified as described [human GST-SF3B2 (401–550);^13^*C. elegans* SFTB-2;^48^ human histone H2B (New England Biolabs, M2505S)]. *Xenopus laevis* histone H2B was expressed in *E. coli* and purified similarly to what was previously reported by Luger et al.^72^

Inhibitors used were 5′-deoxy-5′-(methylthio)adenosine (methylthioadenosine, MTA; Sigma, cat. no. D5011) and EPZ015666 (APEx Bio, Cat. No. B4989).

### In Vitro Methylation Reactions with MTA and EPZ015666

Reactions consisting of approximately 1 μg of enzyme (human or *C. elegans* GST-tagged enzymes PRMT9 and PRMT7), reacted with approximately 5 μg of substrate [for human PRMT9, human GST-SF3B2 401–550 fragment; *C. elegans* PRMT9 with *C. elegans* GST-SFTB-2 (99–248) fragment, or recombinant human histone H2B from New England Biolabs (M2505S)], were incubated for the indicated time in reaction buffer {50 mM potassium HEPES buffer, 10 mM NaCl, 1 mM dithiothreitol (DTT), pH 8.0 with 0.7 μM *S*-adenosyl-l-[methyl-^3^H]methionine (^3^H-AdoMet, PerkinElmer Life Sciences, 82.7 Ci/mmol, 0.55 mCi/mL in 10 mM H_2_SO_4_/EtOH [9:1 (v/v)])} in a final reaction volume of 60 μL. Each reaction was incubated at the corresponding optimal reaction temperature (37 °C for human PRMT9, 25 °C for *C. elegans* PRMT9, and 15 °C for *C. elegans* and human PRMT7 enzymes. MTA was dissolved in water, and a wavelength scan was taken to determine the final concentration using the extinction coefficient. The final concentrations used are indicated in the figure legends. EPZ015666 was dissolved in DMSO to a final concentration of 13.04 mM, and further dilutions were made in DMSO to achieve the final concentrations used as indicated in the figure legends. EML734, EML736, and EML709 were also dissolved in DMSO, and further dilutions were made in DMSO for the working stocks. For controls, no inhibitor reactions were created by the addition of the respective solvent (water for MTA and DMSO for EPZ015666 and EML inhibitors).

### Detection of Inhibition Activity after SDS-PAGE

After the reaction incubations with the various concentrations of inhibitors, the reactions were quenched by adding 0.2 volume of 5× SDS sample loading buffer, and subsequently, the reactions were run on a 12.6% polyacrylamide Tris gel. To collect the radioactive signal, the gels were then treated in the same way as previously described.^48^

### Analysis of Densitometric Data

After various exposures were collected to ensure linear detection, densitometry analysis was done using ImageJ software, and data were plotted as normalized activity to the no inhibitor controls.

### Molecular Modeling and Molecular Dynamics Methods

Docking experiments were attained for compound **1a** on the X-ray structure of the human PRMT9 in complex with the adenosine-based inhibitor (MT556, PDB code 7RBQ)^59^ and on the Alphafold structure of the human PRMT7 enzyme. Before these receptor structures could be utilized in docking calculations, they required preparation using the Protein Preparation Wizard^73,74^ utility within the Maestro software package.^75^ The receptor structures were prepared by assigning bond orders, adding hydrogens, and generating physiological pH states using the EPIK tool. Subsequently, the “Minimize and Delete Waters” tool was employed to minimize the overall protein structures, with heavy atoms restrained and all water molecules removed. In order to prepare **1a** for docking calculations, a separate tool within the Schrödinger software suite known as “LigPrep” was utilized. Specifically, all the hydrogen atoms were added, all the tautomeric states were generated, and the specified chiralities were retained. The AUTODOCK-GPU (AD4-GPU)^58^ is an accelerated version of AutoDock 4.2.6 able to increase docking calculation speed.^76^ Before launching all docking calculations, for every ligand/receptor complex, a 60 Å × 60 Å × 60 Å with a 0.375 Å spacing grid was calculated around the binding site for ligand atom types using AutoGrid4. In the context of performing docking calculations, a crucial preliminary step involves the conversion of ligand structures from the PDB to the PDBQT format, which is required by AutoDock software. This conversion is typically accomplished using the AutoDock Tools (ADT) utility known as “prepare_ligand4.py”. All docking calculations were accomplished on our GPUs (NVIDIA RTX A6000 and Quadro RTX 8000). To improve the accuracy and speed of the docking calculations, a heuristic parameter was incorporated into the AutoDock GPU algorithm. This parameter guides the search algorithm toward the most promising solutions based on previous docking experiments. During the automated docking process, only “.xml” output files were generated to reduce the amount of storage required and simplify the data analysis. 200 independent docking simulations were carried out for each docking experiment to ensure the comprehensive exploration of the conformational space and enhance the likelihood of identifying potential drug candidates. Finally, a .csv file was created from .xml files, returning for **1a** the lowest binding free energy, number of runs, mean binding energy, and numbers in the lowest energy cluster.

The latter conformation was considered for the MD simulations. We conducted all-atom molecular dynamics (MD) simulations using the Desmond module^60^ of the Schrödinger software package to study the **1a**/hPRMT9 and **1a**/hPRMT7 complexes. To set up the initial system for the MD calculation, we utilized the system builder panel. The complexes were placed within a parallelepiped box and solvated with TIP3P water models.^77^ To neutralize the system’s charge, Na^+^ ions were added. The equilibration of the systems was carried out using the *NPT* ensemble following the default Desmond protocol, which involved a total of eight steps. The first seven steps were short simulations that gradually increased the temperature and decreased restraints on the solute, aiming to reach an equilibrated state. Subsequently, the equilibrated systems underwent a 500 ns MD production run under periodic boundary conditions (PBC) and the *NPT* ensemble, utilizing the OPLSe force field.^78^ The simulation was conducted at 1 atm pressure and a temperature of 300 K. To maintain these conditions, a Martyna–Tobias–Klein barostat^79^ and a Nose-Hoover chain thermostat^80^ were employed.

### Radioisotope-Based IC_50_ Profiling against PRMTs

The effects of compounds **1i**,**j** on the catalytic activity of PRMT1, PRMT3, PRMT4, PRMT5, PRMT6, PRMT7, and PRMT8 were determined with a HotSpot PRMT activity assay by Reaction Biology Corporation (Malvern, PA, USA) according to the company’s standard operating procedure.^81,82^ Briefly, the full-length human recombinant proteins PRMT1 (residues 2–371, C-terminus; with an N-terminal GST-tag; *M*_W_ = 68.3 kDa; Genbank Accession # NM_001536) or PRMT3 (residues 2–531, C-terminus; with an N-terminal His-tag; *M*_W_ = 62.0 kDa; Genbank Accession # NM_005788), or PRMT4 (residues 2–608, C-terminus; with an N-terminal GST-tag; *M*_W_ = 91.7 kDa; Genbank Accession # NM_199141), or PRMT5/MEP50 complex^83,84^ (residues PRMT5 2–637, C-terminus, and MEP50 2–342, C-terminus; with an N-terminal FLAG-tag, PRMT5, or His-tag, MEP50; *M*_W_ = 73.7/39.9 kDa; Genbank Accession # NM_006109, NM_006109), or PRMT6 (residues 2–375, C-terminus; with an N-terminal GST-tag; MW = 67.8 kDa; Genbank Accession # NM_018137), or PRMT7 (residues 2–692, C-terminus; with an N-terminal His-tag; *M*_W_ = 81.7 kDa; Genbank Accession # NM_019023), or ΔN(1–60)-PRMT8^85^ (residues 61–394, C-terminus; with C- and N-terminal His-tags; *M*_W_ = 43.2 kDa; Genbank Accession # NM_019854) were added to a solution of the proper substrate (histone H4 for PRMT1, PRMT3, and PRMT8; histone H3.3 for PRMT4; histone H2A for PRMT5/MEP50; GST-GAR for PRMT6 and PRMT7; final concentration 5 μM) in freshly prepared reaction buffer (50 mM Tris-HCl (pH 8.5), 5 mM MgCl_2_, 50 mM NaCl, 1 mM DTT, 1 mM PMSF, 1% DMSO) and gently mixed. The proper solution of compound **1i**–**j** in DMSO was delivered into the PRMT reaction mixture by using Acoustic Technology (Echo 550, LabCyte Inc. Sunnyvale, CA) in nanoliter range, and incubated for 20 min at room temperature. Then, ^3^H-SAM (final concentration of 1 μM) was delivered into the reaction mixture to initiate the reaction. After incubation for 60 min at 30 °C, the reaction mixture was delivered to filter-paper for detection (as assessed by scintillation). Data were analyzed using Excel and GraphPad Prism 6.0 software (GraphPad Software Inc., San Diego, CA) for IC_50_ curve fits using sigmoidal dose vs response - variable slope (four parameters) equations.

### Selectivity Assay against KMTs

The effects of compound **1j** on the catalytic activity of ASH1L/KMT2H, EZH2/KMT6, G9a/KMT1C, MLL1/KMT2A, SET7/9/KMT7, SMYD3/KMT3E, SUV39H2/KMT1B, and DOT1L/KMT4 were determined with a HotSpot KMT activity assay by Reaction Biology Corporation (Malvern, PA, USA) according to the company’s standard operating procedure.^81,82^ Briefly, the human recombinant ASH1L (residues 2046–2330, with an N-terminal His-tag; *M*_W_ = 35.4 kDa; Genbank Accession # NM_018489), or the human recombinant EZH2-containing five-member polycomb repressive complex 2 (including EZH2 residues 2–746, AEBP2 2–517, EED 2–441, RbAp48 2–425, SUZ12 2–739; all full-length; with N-terminal Flag-tag on EED and N-terminal His-tag on all others; *M*_W_ = 333.8 kDa; Genbank Accession # NM_001203247, NM_001114176, NM_003797, NM_005610, NM_015355), or the human recombinant G9a (residues 913–1193, C-terminus; with an N-terminal His-tag; *M*_W_ = 34.6 kDa; Genbank Accession # NM_006709.3), or the human recombinant MLL1 complex (including MLL1 residues 3745–3969, C-terminus, WDR5 22–334, C-terminus, RbBP5 1–538, C-terminus, ASH2L 2–534, C-terminus, DPY-30 1–99, C-terminus; N-terminal His-tag on all subunits; *M*_W_ = 212.0 kDa; Genbank Accession # NM_005933, NM_017588, NM_005057, NM_001105214, NM_0325742), or the human recombinant SET7/9 (residues 2–366, C-terminus; with a N-terminal GST-tag and a C-terminal His-tag; *M*_W_ = 68.5 kDa; Genbank Accession # NM_030648), or the human recombinant SMYD3 (residues 2–428, C-terminus; C-terminal His-tag; *M*_W_ = 50.1 kDa; Genbank Accession # NM_001167740), or the human recombinant SUV39H2 (residues 46–410, C-terminus; N-terminal fusion protein with a C-terminal His-tag; *M*_W_ = 98.8 kDa; GenBank Accession No. NM_001193424), or the human recombinant DOT1L (residues 1–416; N-terminal GST-tag; *M*_W_ = 80.0 kDa; Genbank Accession # NM_032482) was added to a solution of the proper substrate (oligo nucleosomes for ASH1L, MLL1 complex, and DOT1L, final concentration 0.05 mg/mL; core histone for EZH2 complex and SET7/9, final concentration 0.05 mg/mL; histone H3 for SUV39H2, final concentration 5 μM; histone H3 1–21 for G9a, final concentration 2.5 μM) in freshly prepared reaction buffer (50 mM Tris-HCl (pH 8.5), 5 mM MgCl_2_, 50 mM NaCl, 1 mM DTT, 1 mM PMSF, 1% DMSO) and gently mixed. The proper solution (1 or 10 μM fixed concentrations) of compound **1j** in DMSO was delivered into the KMT reaction mixture by using Acoustic Technology (Echo 550, LabCyte Inc. Sunnyvale, CA) in the nanoliter range and incubated for 20 min at room temperature. Then, ^3^H-SAM (final concentration 1 μM) was delivered into the reaction mixture to initiate the reaction. After incubation for 60 min at 30 °C, the reaction mixture was delivered to filter paper for detection (as assessed by scintillation). SAH^62−64^ or chaetocin (for ASH1L)^65^ was used as reference compounds and tested in 10-dose IC_50_ mode with 3-fold serial dilution starting at 100 μM. No inhibitor control (DMSO) was considered as showing 100% enzyme activity. Data were analyzed using Excel and GraphPad Prism 6.0 software (GraphPad Software Inc., San Diego, CA). Values obtained for each compound are mean ± SD determined for two separate experiments.

### PRMT9 SPR Experiments

SPR experiments were performed on a Biacore T200 biosensor (Cytiva). PBS buffer (phosphate buffered saline, pH 7.5) supplemented with 0.05% Tween-20 was used as the running buffer. Full-length recombinant PRMT9 (2–285, BPS Bioscience, # BPS-79124) was covalently immobilized on the carboxymethylated surface of a Series S Sensor Chip CM5 by amine coupling. In detail, 50 μg/mL of protein in phosphate buffer (40 mM NaH_2_PO_4_/Na_2_HPO_4_ pH 8.0, 110 mM NaCl, 2.2 mM KCl, 0.04% Tween-20, and 2 mM TCEP) was preconcentrated on the surface with 10 mM of sodium acetate pH 4.5 after surface activation with EDC/NHS (1:1) was covalently immobilized using the running buffer 1X HBS – 0.05% Tween20 at a flow rate of 10 μL/min to obtain densities of 8.3 kRU. The compound of interest was diluted in PBS supplemented with 0.05% Tween-20 and injected over the active and reference cells at 10 different concentrations (2-fold dilution series) from 25 μM to 0.05 μM, keeping a final 2% DMSO concentration, using the multicycle modality. Binding experiments were performed at 25 °C by using a flow rate of 30 μL/min, with 90 s of monitoring of association and 180 s of monitoring of dissociation. Regeneration of the surfaces was performed, when necessary, by a 10 s injection of 5 mM NaOH. The sensorgrams obtained at the 10 concentrations of the compound were first corrected taking advantage of the solvent correction performed by the instrument (correction range from 1.5 to 2.8% DMSO), and then they were double-referenced. The corrected sensorgrams were fitted simultaneously by kinetic analysis using the 1:1 Langmuir model of the BIAevaluation software to obtain equilibrium dissociation constants (*K*_D_) and kinetic dissociation (*k*_off_) and association (*k*_on_) constants. The curve-fitting efficiency was evaluated by the chi-square (χ^2^). The χ^2^ value denotes the fitting degree between the estimative and experimental curves.

### PAINS Analysis

Compounds **1i** and **1j** were analyzed for known classes of assay interference compounds.^86^ All derivatives were not recognized as PAINS according to the SwissADME web tool (http://www.swissadme.ch),^87^ the Free ADME-Tox Filtering Tool (FAF-Drugs4) program (http://fafdrugs4.mti.univ-paris-diderot.fr/),^70^ and the “False Positive Remover” software (http://www.cbligand.org/PAINS/);^88^ neither were they recognized as aggregators according to the software “Aggregator Advisor” (http://advisor.bkslab.org/).^89^

### Western Blotting Methods

To test the effect of compounds **1a**, **1c**, **1e**, and **1f** on PRMT9 activity, MCF cells or MDA-MB-436 cells were treated with 4 candidate inhibitors at indicated concentrations. After 72 h, cells were harvested in ice-cold PBS and were lysed in radio immune-precipitation assay (RIPA) buffer (50 mM Tris [pH 8.0], 150 mM NaCl, 1% Triton X-100, 0.5% sodium deoxycholate, 0.1% SDS, 2 mM EDTA, and protease inhibitors). For immunoblotting, an equal amount of each sample was resolved by sodium dodecyl sulfate-polyacrylamide gel electrophoresis (SDS-PAGE) and transferred to a polyvinylidene difluoride (PVDF) western membrane. Following blocking with 5% nonfatty milk in PBS-T, membranes were incubated with indicated primary antibodies at 4 °C overnight. The HRP-conjugated secondary antibodies were used against respective primary antibodies. The antibody–antigen complexes were visualized by the chemiluminescence method by using X-ray films.

### Proteomic Analysis

Control and **1a**- and **1j**-treated HEK293T cell pellets were suspended in 200 μL of 8 M urea/50 mM ammonium bicarbonate (AmBic, pH 8.5), 0.5% w/v sodium deoxycholate, and 1× protease inhibitor cocktail (GeneSpin); the suspensions were lysed through sonication (Vibra cell; SONICS; 1 min, 30% amplitude, 9.9 s pulses) and then centrifuged (21,000 rcf, 18 °C, 30 min). Protein concentration was determined through Bradford assay (Bio-Rad).

For each sample, 300 μg of proteins were submitted to our optimized *in-solution* digestion protocol,^90^ reducing disulfide bridges with 1,4-dithiothreitol (DTT, 10 mM) for 1 h at 25 °C and 800 rpm and then alkylating them with iodoacetamide (IAA, 20 mM) for 30 min, at 25 °C and 800 rpm, in the dark. Then, IAA was quenched with 10 mM DTT, urea was diluted to 1 M with 50 mM AmBic and a trypsin/LysC solution (Promega, Madison, Wisconsin) was added at the enzyme to proteins ratio of 1:100 w/w, overnight at 37 °C.

The peptidic mixtures were then desalted through Sep-Pak C18 1 cc (50 mg) cartridges (Waters, Milford, USA), as reported by the manufacturer, and redissolved in 10% TFA for the subsequent analysis.

1.5 μg sample of each digest was analyzed on an Orbitrap Q-Exactive Classic Mass Spectrometer (ThermoFisher Scientific, Bremen, Germany) coupled to an UltiMate 3000 Ultra-High-Pressure Liquid Chromatography (UHPLC) system (ThermoFisher Scientific, Bremen, Germany), equipped with an EASY-Spray PepMAP RSLC C18 column (3 μm, 100 Å, 75 μm × 50 cm, ThermoFisher Scientific, Bremen, Germany) at a flow rate of 300 nL/min with the following gradient: 1 min at 3% B, 1 to 100 min to 38% B, 100 to 101 min to 80% B, then held at 80% B for 10 min, and re-equilibrated for 8 min at 3% B (A: 95% H_2_O, 5% CH_3_CN, 0.1% AcOH; B: 95% CH_3_CN, 5% H_2_O, 0.1% AcOH). The mass spectrometer was operated in data-dependent acquisition mode. Full-scan MS spectra were acquired with the scan range 375–1500 *m*/*z*, a full-scan automatic gain control (AGC) target 3e6 at 70,000 resolution, and a maximum injection time of 50 ms. MS2 spectra were generated for up to 8 precursors (normalized collision energy of 28%), and the fragment ions were acquired at a resolution of 17,500 with an AGC target of 1e5 and a maximum injection time of 80 ms. Protein identification and label-free quantification were then achieved through Proteome Discoverer (version 2.4.1.15). A spectral library search (NIST Human Orbitrap HCD Library, 1127970 spectra, September 2016) was performed through MSPepSearch, and then MS/MS spectra were searched by Sequest against a reviewed *Homo sapiens* database (SwissProt, February 2022, 20,594 entries) with the following parameters: trypsin digestion; maximum of 5 missed cleavages; cysteine carboxyamidomethylation as fixed modification; arginine mono- or dimethylation, methionine oxidization, protein N-terminal acetylation and/or demethylation as variable modifications. Mass tolerances were 50 ppm for MS1 and 0.02 Da for MS/MS. Label-free quantification was achieved by using both unique and razor peptides for peptides and protein abundance calculation, and a pairwise ratio-based approach was used to evaluate the EML-treated vs control peptides and protein abundance. For each calculated ratio, a background-based *t-*test was performed.

## Supporting Information Available

Optimization of the PRMT9 AlphaLISA assay, docking studies on PRMT9, results of the selectivity assay against KMTs, and copies of ^1^H NMR,^13^C NMR, and ^19^F NMR spectra of all final compounds (PDF)
The output of the computational studies (PDB) (PDB)
Molecular formula strings (CSV)

## Supplementary Material

jm3c01030_si_001.pdf

jm3c01030_si_002.pdb

jm3c01030_si_003.pdb

## ABBREVIATIONS

AcOEt: ethyl acetate
ACN: acetonitrile
AEBP2: zinc finger protein AEBP2, also known as Adipocyte enhancer-binding protein 2
ALPHA: amplified luminescent proximity homogeneous assay
ASH1L: absent small and homeotic disks protein 1 homologue
ASH2L: Set1/Ash2 histone methyltransferase complex subunit ASH2, also known as absent small and homeotic disks protein 2 homologue
CA150: also known as TCERG1
CARM1: coactivator-associated arginine methyltransferase 1
CBP: CREB-binding protein
CREB: cAMP response element-binding protein
DEPTQ: distortionless enhancement by polarization transfer quaternary
DOT1L: DOT1-like (disruptor of telomeric silencing 1-like)
DPY30: protein dpy-30 homologue also known as Dpy-30-like protein
EDC: 1-ethyl-3-(3-(dimethylamino)propyl)carbodiimide
ECL: enhanced chemiluminescence
EED: Polycomb protein EED, also known as Embryonic ectoderm development protein
ELAV: embryonic lethal, abnormal vision, Drosophila
ELAVL1: ELAV like RNA binding protein 1
ELAVL4: ELAV like RNA binding protein 4
EZH2: enhancer of zeste homologue 2
GAR: glycine- and arginine-rich motif
GST: glutathione*S*-transferase
HNRNPA1: heterogeneous nuclear ribonucleoprotein A1
HSP70: heat shock 70 kDa protein 1B
HuD: human antigen D, also known as ELAVL4
HuR: human antigen R, also known as ELAVL1
LFQ: label-free quantification method in mass spectrometry
LNCaP: lymph node carcinoma of the prostate cell line
MAVS: mitochondrial antiviral signaling protein
MED12: mediator of RNA polymerase II transcription subunit 12
MLL1: histone-lysine *N*-methyltransferase 2A, also known as myeloid/lymphoid or mixed-lineage leukemia protein 1
MTA: methylthioadenosine
MTT: 3-(4,5-dimethylthiazol-2-yl)-2,5-diphenyltetrazolium bromide
NFIB-Me: nuclear factor 1 B-type
p300: E1A-associated protein of 300 kDa
P_app_: apparent permeability
PABP1: poly(A)-binding protein 1
Pbf: 2,2,4,6,7-pentamethyldihydrobenzofuran-5-sulfonyl
PBST: phosphate buffered saline with Tween 20
PMSF: phenylmethylsulfonyl fluoride
PRMT: protein arginine methyltransferase
PRMT1: protein arginine methyltransferase 1
PRMT3: protein arginine methyltransferase 3
PRMT4: protein arginine methyltransferase 4
PRMT5: protein arginine methyltransferase 5
PRMT6: protein arginine methyltransferase 6
PRMT7: protein arginine methyltransferase 7
PRMT8: protein arginine methyltransferase 8
PVDF: polyvinylidene difluoride
RBAP48: histone-binding protein RBBP4, also known as retinoblastoma-binding protein p48
RBBP5: retinoblastoma-binding protein 5
SAH: S-5′-adenosyl-l-homocysteine
SAM: *S*-adenosyl-l-methionine
SD: standard deviation
SRC-3: steroid receptor coactivator-3
SET: suppressor of variegation 3–9 enhancer-of-zeste trithorax
SET7/9: SET domain-containing protein 7
SETD8: SET domain-containing protein 8, also known as PR/SET domain-containing protein 07
siRNA: small interference ribonucleic acid
SF3B2: splicing factor 3B subunit 2 also known as spliceosome-associated protein 145, SAP145
SMYD3: SET (Suppressor of variegation Enhancer of Zeste Trithorax) and MYND (myeloid-Nervy-DEAF-1) domain-containing protein 3
SUV39H2: suppressor of variegation 3–9 homologue 2 also known as Su(var.)3–9 homologue 2
SUV420H1-TV2: Suppressor of variegation 4–20 homologue 1 -transcription variant 2
SUZ12: polycomb protein SUZ12 also known as suppressor of zeste 12 protein homologue
TCERG1: transcription elongation regulator 1
WDR5: WD repeat-containing protein 5
