# Supplementary material for: Identification of a Protein Arginine Methyltransferase 7 (PRMT7)/Protein Arginine Methyltransferase 9 (PRMT9) Inhibitor
Source: J Med Chem. 2023 Aug 10;66(19):13665–83. doi: 10.1021/acs.jmedchem.3c01030 (PMC10578352; doi:10.1021/acs.jmedchem.3c01030)
Supplement: Supplementary file 1 — jm3c01030_si_001.pdf [file jm3c01030_si_001.pdf]

Supporting Information

Identification of a Protein Arginine Methyltransferase 7 (PRMT7)/Protein Arginine Methyltransferase 9 (PRMT9) Inhibitor

Alessandra Feoli,[‡,∥] Giulia Iannelli,[‡,ʃ,†,∥] Alessandra Cipriano,[‡] Ciro Milite,[‡] Lei Shen,[△] Zhihao Wang,[△] Andrea Hadjikyriacou,[§] Troy L. Lowe,[§] Cyrus Safaeipour,[§] Monica Viviano,[‡] Giuliana Sarno,[‡,ʃ] Elva Morretta,[∩] Maria Chiara Monti,[∩] Yanzhong Yang,[△] Steven G. Clarke,[§] Sandro Cosconati,[,¥] Sabrina Castellano,[*,‡] and Gianluca Sbardella[*,‡]*

[‡]Department of Pharmacy, Epigenetic Med Chem Lab, [∩]Department of Pharmacy, ProteoMass Lab, and [ʃ]PhD Program in Drug Discovery and Development, University of Salerno, via Giovanni Paolo II 132, I-84084 Fisciano (SA), Italy

[§]Department of Chemistry and Biochemistry, and the Molecular Biology Institute, University of California, Los Angeles, California 90095, USA

[△]Department of Cancer Genetics and Epigenetics, Beckman Research Institute, City of Hope National Cancer Center, Duarte, California 91010, USA

[¥]DiSTABiF, University of Campania "Luigi Vanvitelli", Via Vivaldi 43, 81100 Caserta, Italy

*S.C.: E-mail, sandro.cosconati@unicampania.it; *S.C.: E-mail, scastellano@unisa.it; *G.S.: E-mail, gsbardella@unisa.it

Table of Contents:

Figure S1: Optimization of the ALPHA-based screening protocol for PRMT9 S2

Figure S2: Binding mode of **1a** in complex with PRMT7 S3

Figure S3: Inhibitory activity profiles of compound **1j** against a panel of KMTs S4

Table S1. Inhibitory activity values of compound **1j** against a panel of KMTs S4

^{1}H-NMR and ^{13}C-NMR spectra of compound **1i** S5–S6

^{1}H-NMR, ^{13}C-NMR, and ^{19}F spectra of compound **1j** S7–S9

HPLC traces of compounds **1i–1j** S10–S11

Figure S1. Optimization of the ALPHA-based screening protocol for PRMT9

a)

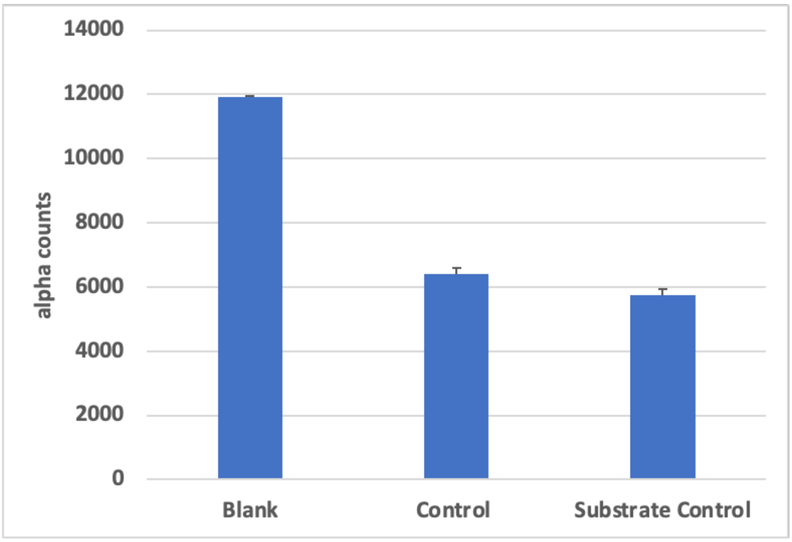

b)

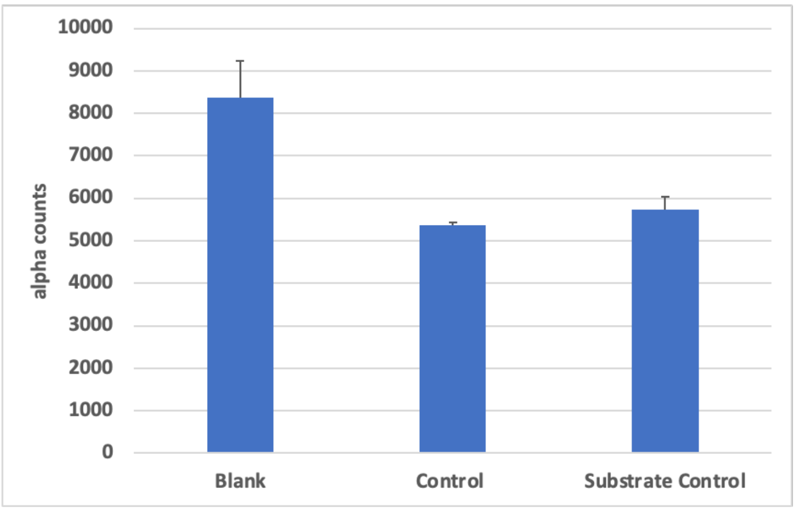

c)

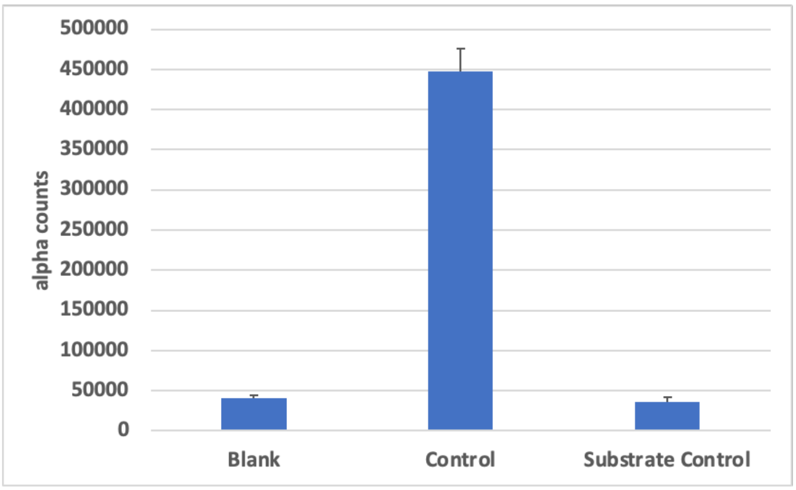

Figure S2

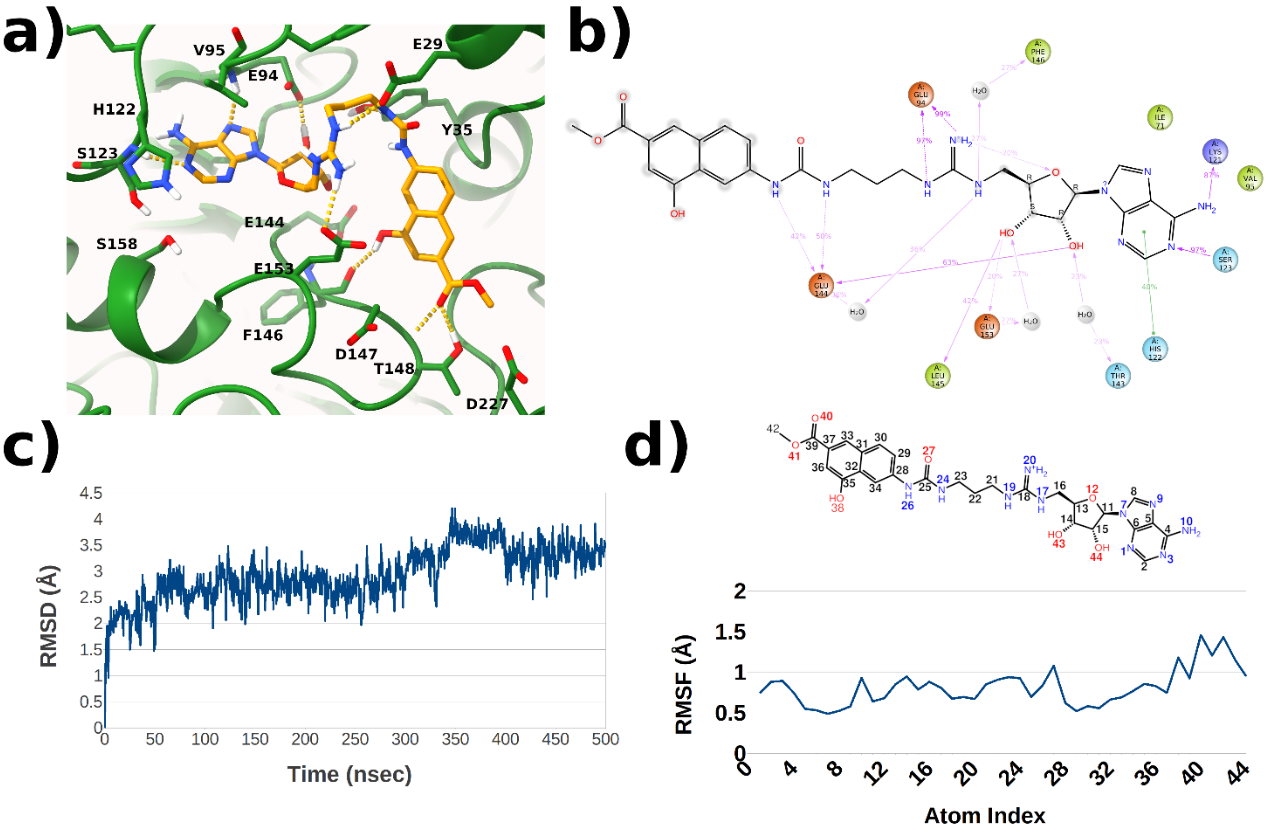

Binding mode of **1a** in complex with PRMT7 3D structure as predicted by docking calculations (a) and 2D representation of ligand/protein interactions through the 500 ns long MD simulation (b). The ligand and enzyme are represented as orange and green sticks and ribbons, respectively. L-RMSD (c) and L-RMSF (d) plots obtained from the analysis of MD simulations.

Figure S3

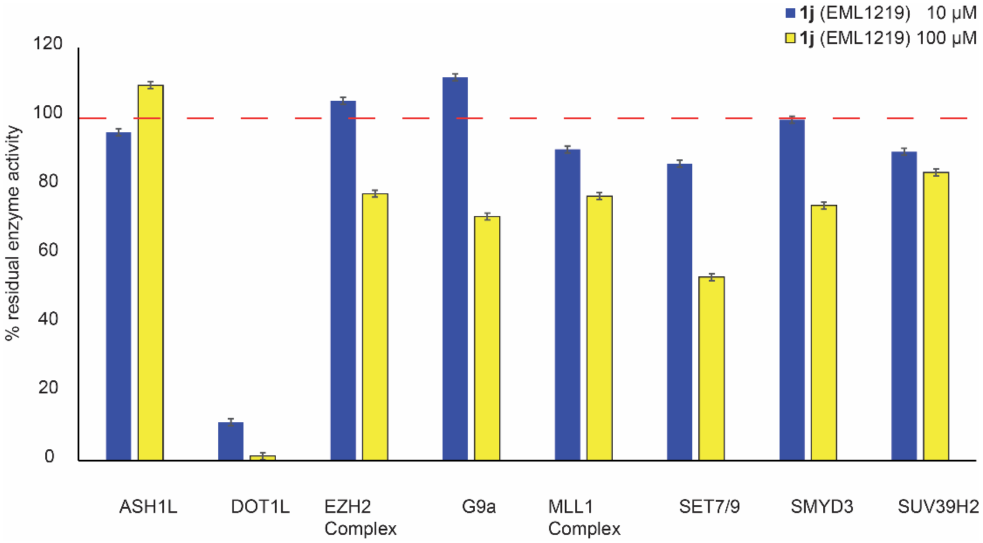

Inhibitory activity profiles of compound **1j** towards ASH1L, DOT1L, EZH2, G9a, MLL1, SET7/9, SMYD3, and SUV39H2 at two different fixed concentrations (10 and 100 μM).

Table S1. Inhibitory activity values of compound **1j** against a panel of KMTs

	**% enzyme residual activ. after treatment with 1j[a,b,]**		**ref. cmpd. IC_{50}(μM)[b,c]**	
	10 μM	100 μM	SAH	chaetocin
**ASH1L**	95.38±1.49	109.06±3.80	-	0.025
**DOT1L**	11.21±0.88	1.35±0.17	0.20	-
**EZH2**	104.55±0.99	77.56±3.43	43.7	-
**G9a**	111.35±4.30	70.93±0.31	1.39	-
**MLL1**	90.45±2.83	76.86±3.96	0.30	-
**SET7/9**	86.32±1.40	53.33±0.38	70.4	-
**SMYD3**	99.09±1.41	74.15±1.80	15.9	-
**SUV39H2**	89.74±1.08	83.76±0.22	37.3	-

[a]Compounds were tested at 10 and 100 μM fixed concentrations; values obtained for each compound are mean ± SD determined for two separate experiments.in duplicate; [b]values were obtained in a radioisotope-based filter assay, using 0.05 mg/mL oligo nucleosomes (for ASH1L, DOT1L, and MLL1 complex), 0.05 mg/mL core histone (for EZH2 complex and SET7/9), 5 μM histone H3 (for SUV39H2) or 2.5 μM histone H3 1–21 (for G9a) as substrate and *S*-adenosyl-L-[methyl-^{3}H]methionine (1 μM) as methyl donor; [c]reference compounds were tested in 10-dose IC_{50} mode with 3-fold serial dilution starting at 10 or 100 μM; data were analyzed using Excel and GraphPad Prism 6.0 software (GraphPad Software Inc., San Diego, CA) for IC_{50} curve fits using sigmoidal dose *vs.* response - variable slope (four parameters) equations.

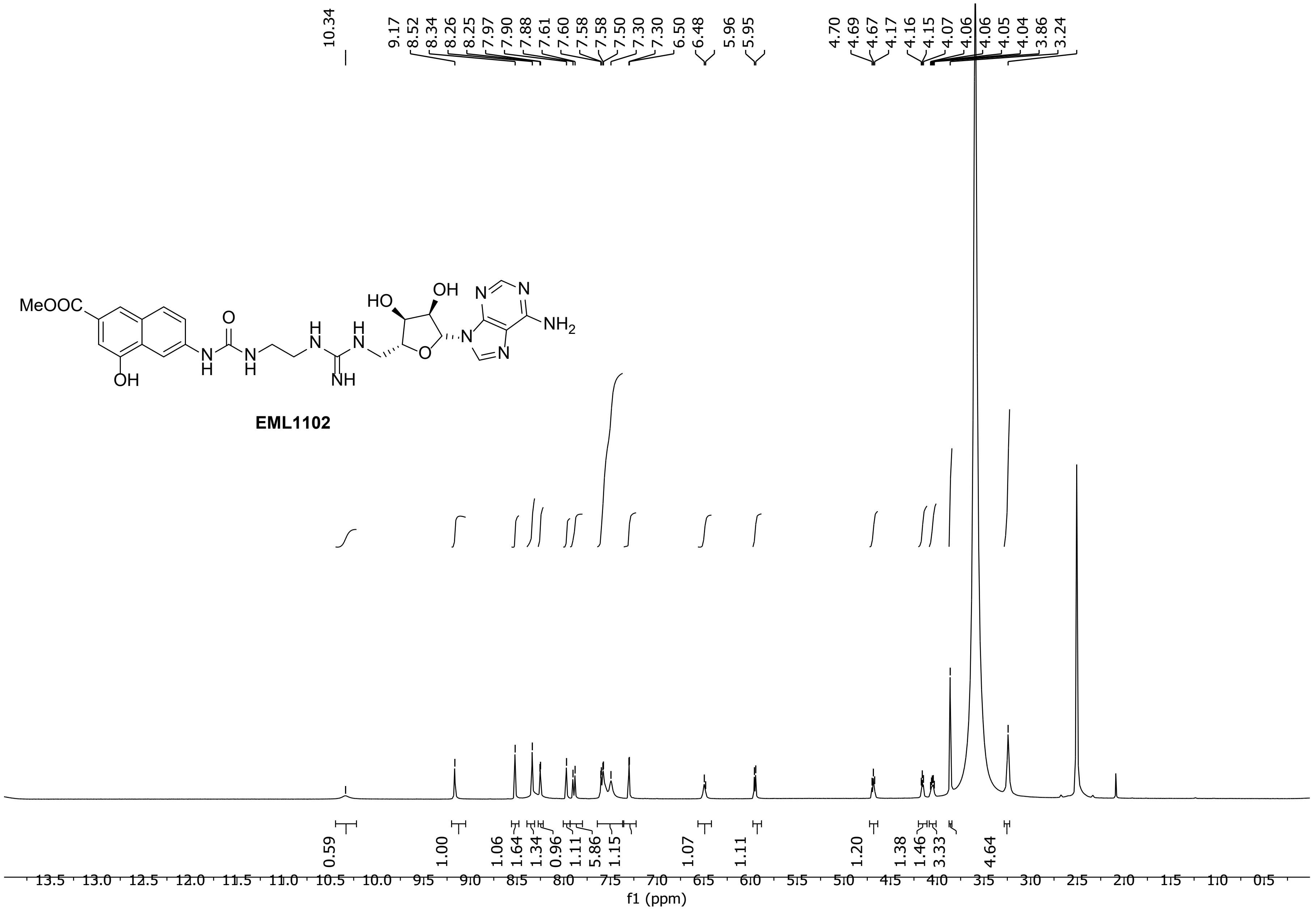
10.34
9.17 8.52 8.34 8.26 8.25 7.97 7.90 7.88 7.61 7.60 7.58 7.58 7.50 7.30 7.30 6.50 6.48 5.96 5.95
4.70 4.69 4.67 4.17 4.16 4.15 4.07 4.06 4.06 4.05 4.04 3.86 3.24
MeOOC
O
OH
HO
OH
N
NH2
NH
EML1102
0.59 1.00 1.06 1.64 1.34 0.96 1.11 5.86 1.15 1.07 1.11 1.20 1.38 1.46 3.33 4.64
13.5 13.0 12.5 12.0 11.5 11.0 10.5 10.0 9.5 9.0 8.5 8.0 7.5 7.0 6.5 6.0 5.5 5.0 4.5 4.0 3.5 3.0 2.5 2.0 1.5 1.0 0.5
f1 (ppm)

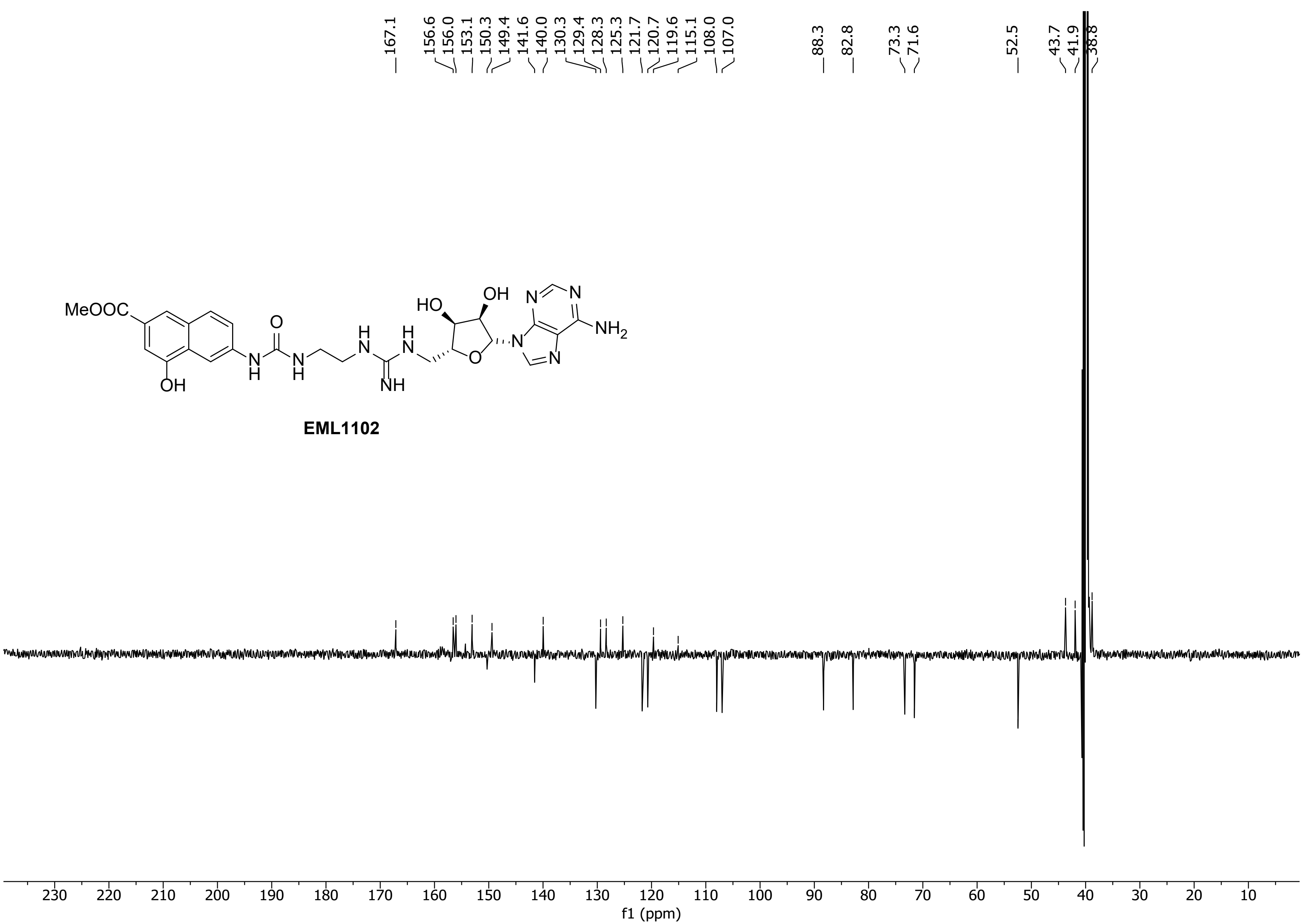
167.1
156.6
156.0
153.1
150.3
149.4
141.6
140.0
130.3
129.4
128.3
125.3
121.7
120.7
119.6
115.1
108.0
107.0
88.3
82.8
73.3
71.6
52.5
43.7
41.9
38.8
MeOOC
OH
N H
N H
H N
H N
NH
HO
OH
O
N
N
N
N
N
NH_2
EML1102
230 220 210 200 190 180 170 160 150 140 130 120 110 100 90 80 70 60 50 40 30 20 10
f1 (ppm)

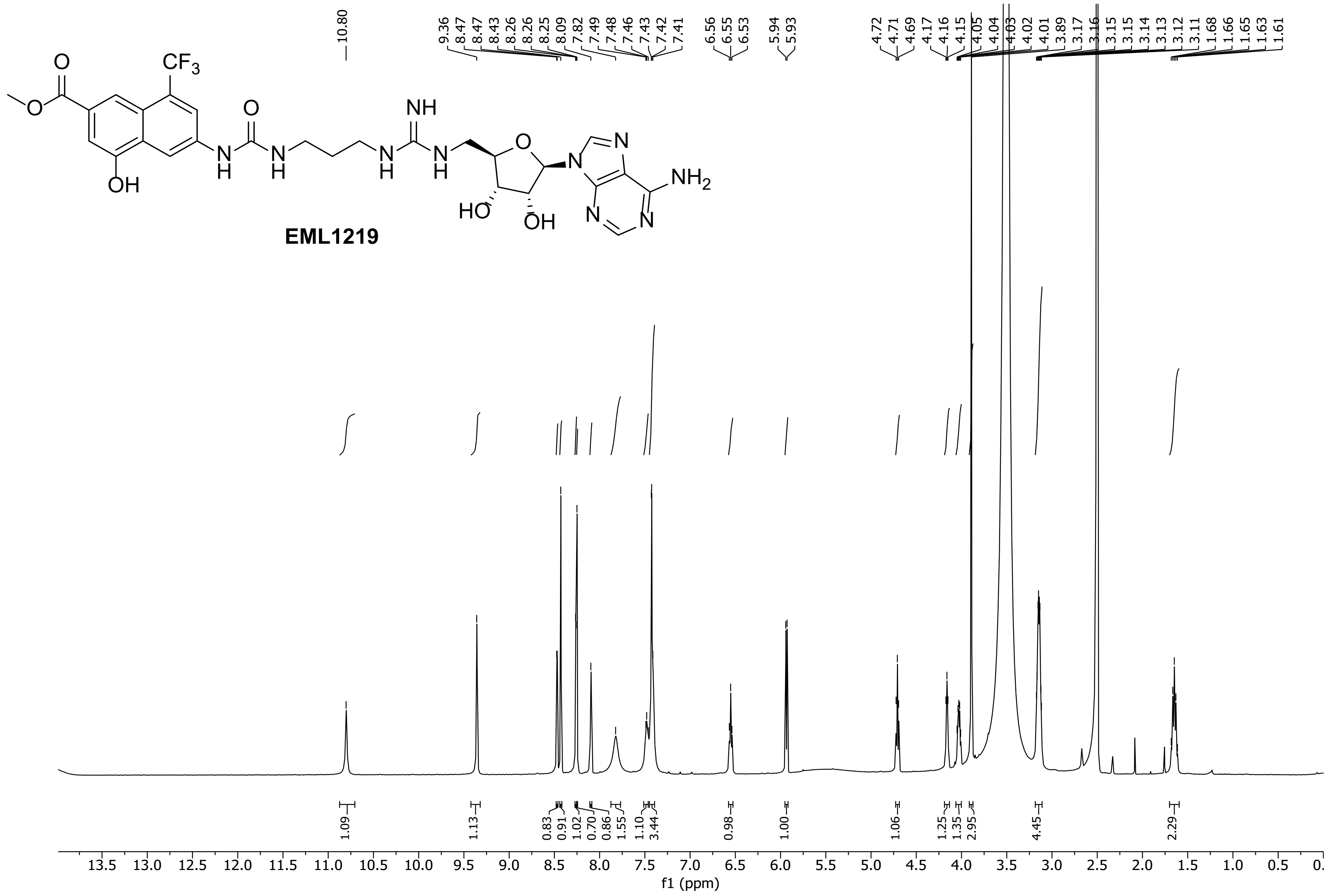
EML1219
CF3
NH
NH2
OH
HO
10.80
9.36
8.47
8.47
8.43
8.26
8.26
8.25
8.09
7.82
7.49
7.48
7.46
7.43
7.42
7.41
6.56
6.55
6.53
5.94
5.93
4.72
4.71
4.69
4.17
4.16
4.15
4.05
4.04
4.03
4.02
4.01
3.89
3.17
3.16
3.15
3.15
3.14
3.13
3.12
3.11
1.68
1.66
1.65
1.63
1.61
1.09
1.13
0.83
0.91
1.02
0.70
0.86
1.55
1.10
3.44
0.98
1.00
1.06
1.25
1.35
2.95
4.45
2.29
13.5 13.0 12.5 12.0 11.5 11.0 10.5 10.0 9.5 9.0 8.5 8.0 7.5 7.0 6.5 6.0 5.5 5.0 4.5 4.0 3.5 3.0 2.5 2.0 1.5 1.0 0.5 0.
f1 (ppm)

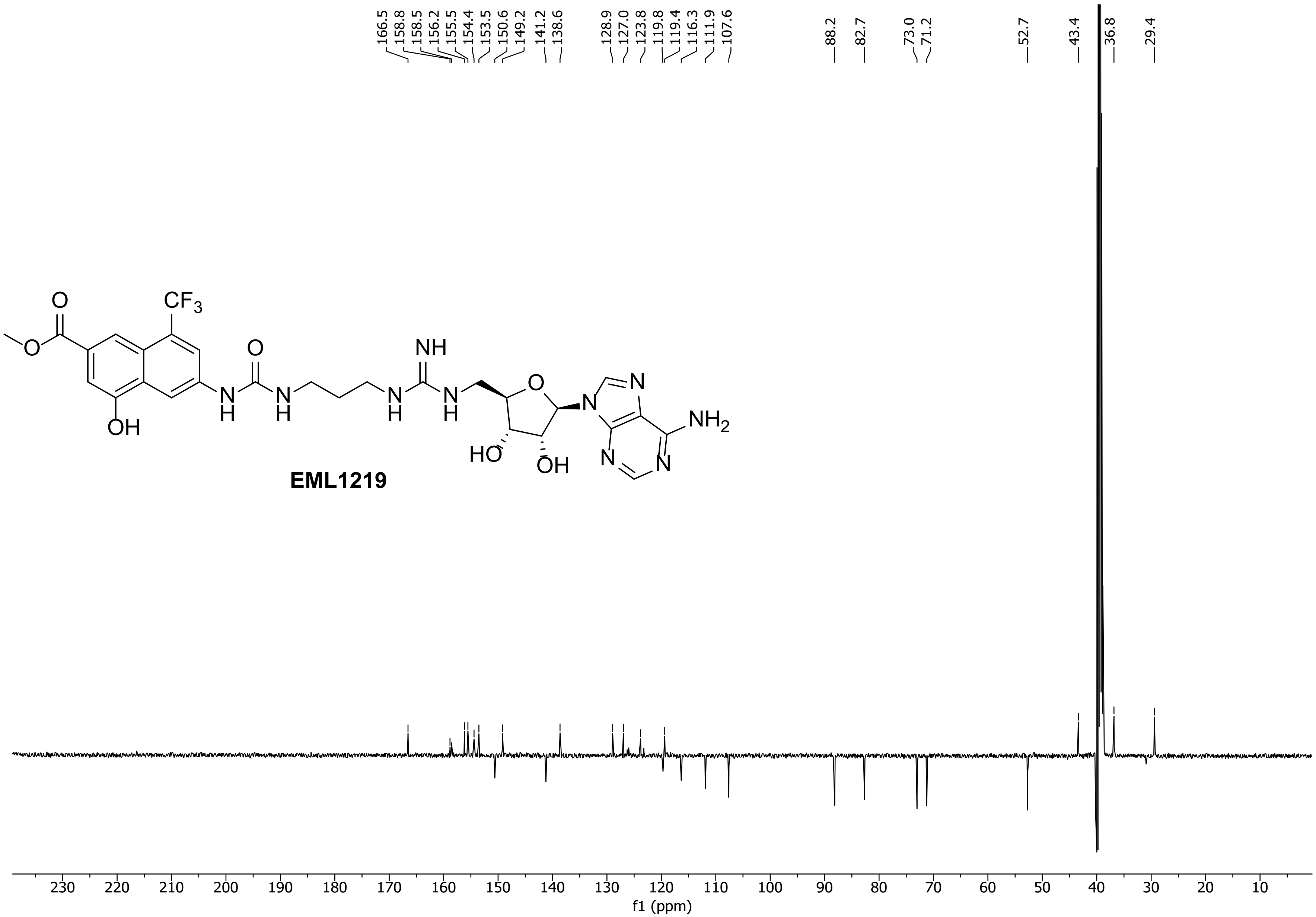

166.5
158.8
158.5
156.2
155.5
154.4
153.5
150.6
149.2
141.2
138.6
128.9
127.0
123.8
119.8
119.4
116.3
111.9
107.6
88.2
82.7
73.0
71.2
52.7
43.4
36.8
29.4
EML1219
230 220 210 200 190 180 170 160 150 140 130 120 110 100 90 80 70 60 50 40 30 20 10
f1 (ppm)

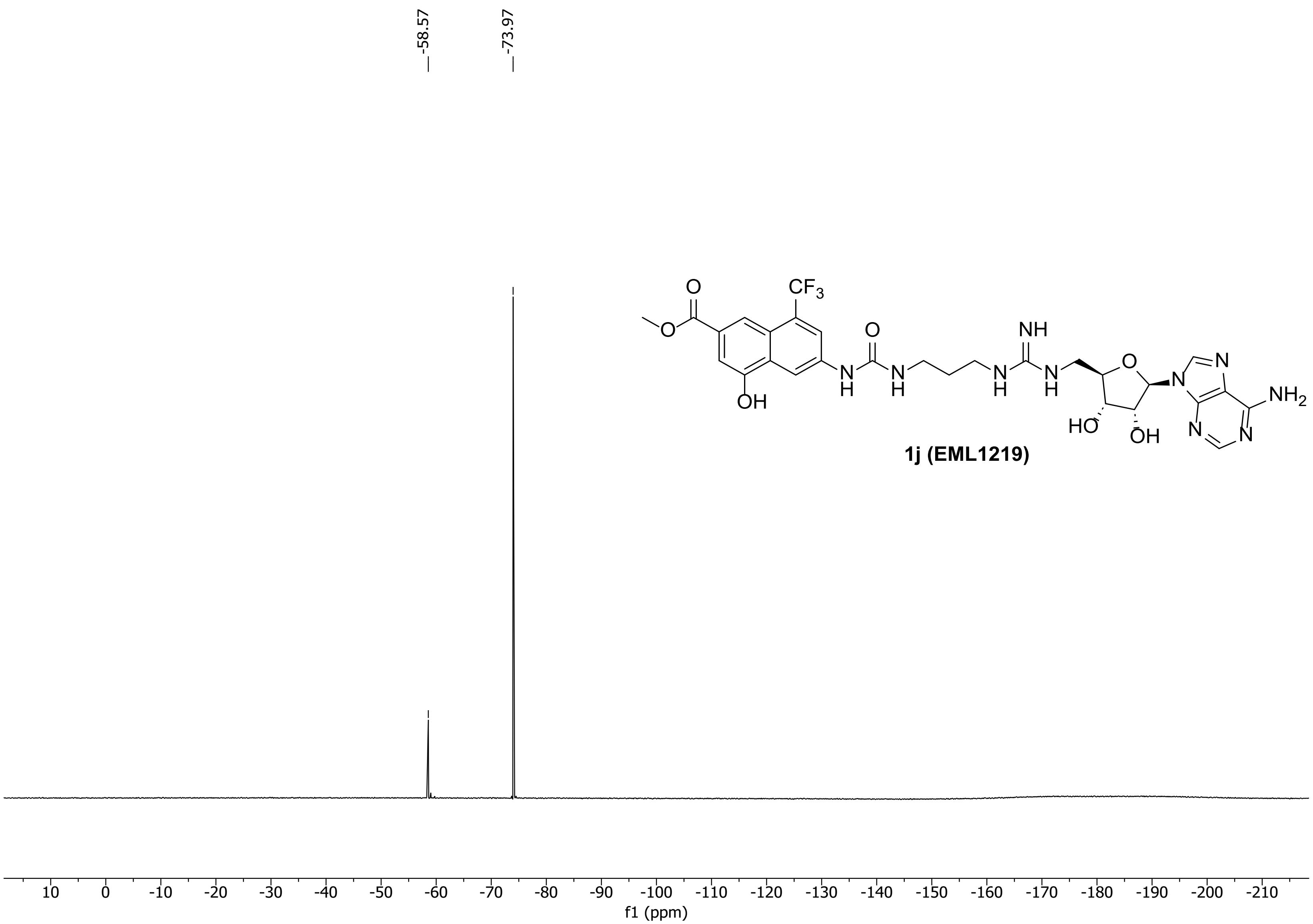
-58.57
-73.97
CF3
O
OH
NH
N H
NH2
HO
1j (EML1219)
10 0 -10 -20 -30 -40 -50 -60 -70 -80 -90 -100 -110 -120 -130 -140 -150 -160 -170 -180 -190 -200 -210
f1 (ppm)

HPLC traces of **1i** (EML1102)

SHIMADTZU HPLC ANALYSIS REPORT

Data File Name : 1i (EML1102).lcd

Chromatogram 220 nm

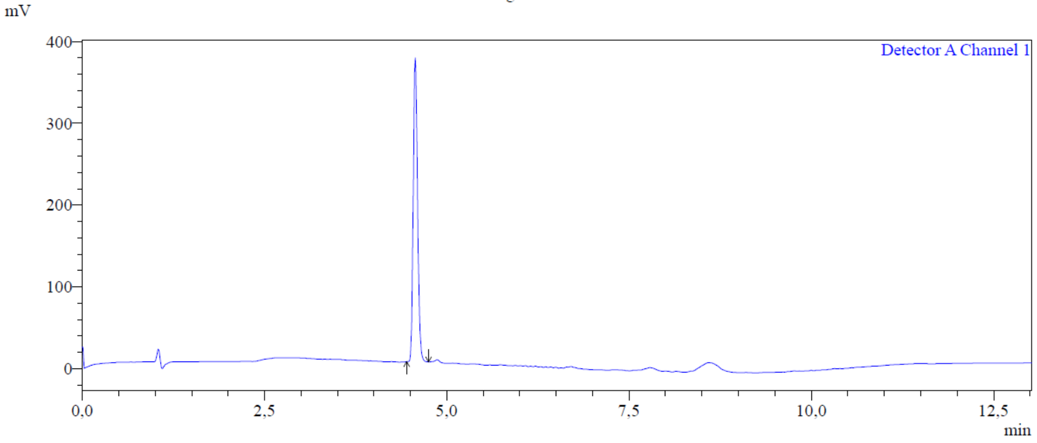

Peak Table

Detector A Channel 1 220nm

Peak#	Ret. Time	Height	Area	Area%
1	4,563	371970	1539969	100,000
Total		371970	1539969	100,000

Chromatogram 254 nm

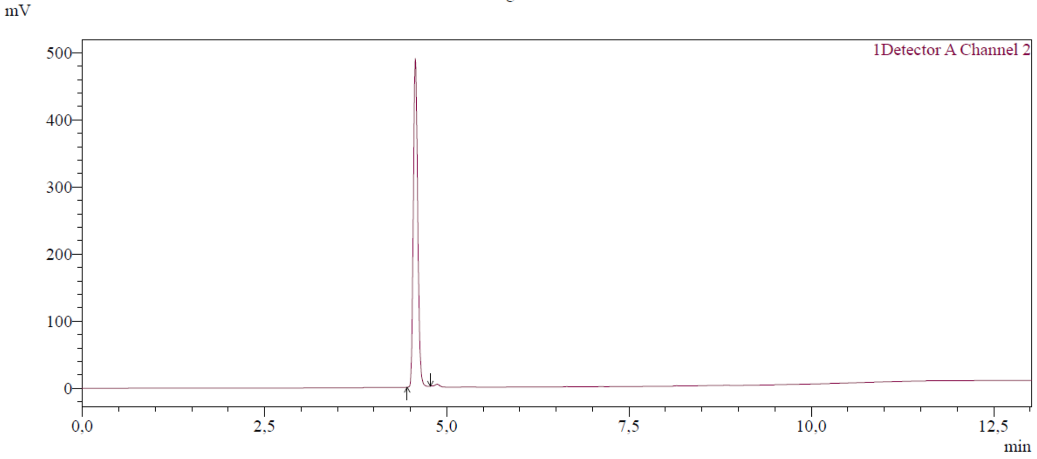

Peak Table

Detector A Channel 2 254nm

Peak#	Ret. Time	Height	Area	Area%
1	4,565	489947	2031642	100,000
Total		489947	2031642	100,000

HPLC traces of **1j** (EML1219)

SHIMADTZU HPLC ANALYSIS REPORT

Data File Name : 1j (EML1219).lcd

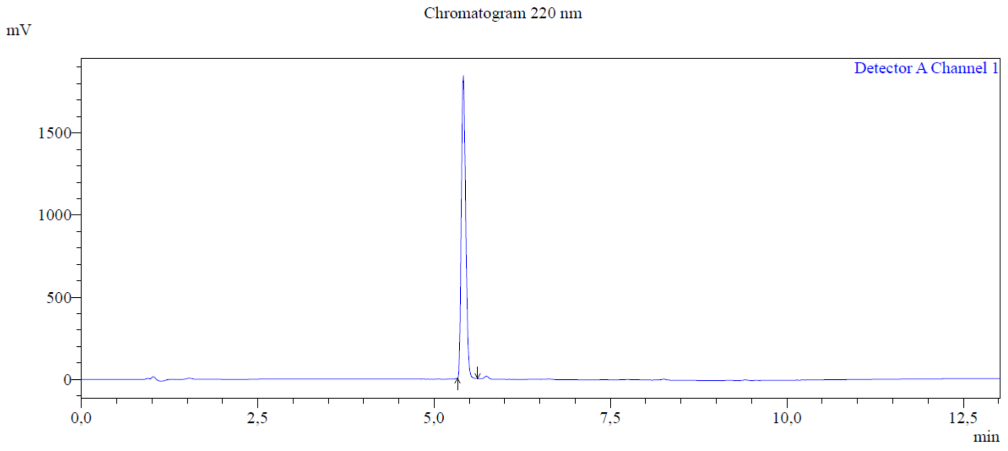

Peak Table

Detector A Channel 1 220nm

Peak#	Ret. Time	Height	Area	Area%
1	5,411	1844671	7968876	100,000
Total		1844671	7968876	100,000

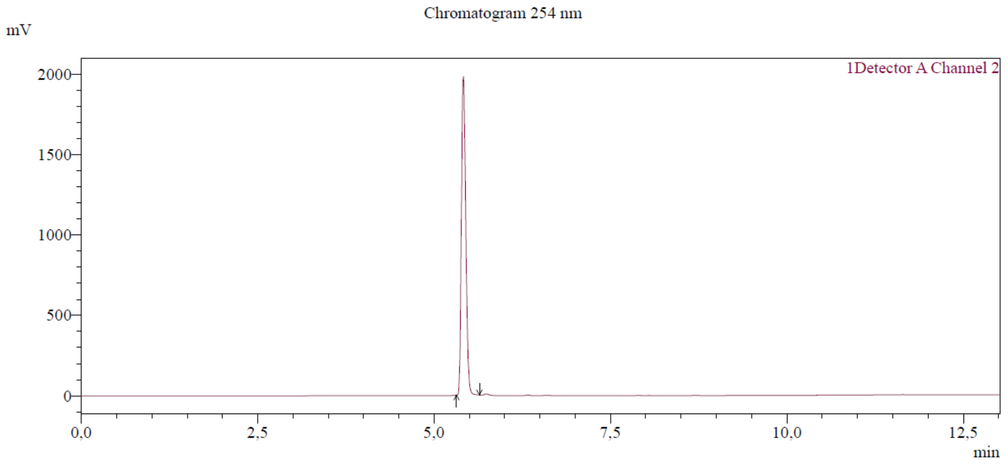

Peak Table

Detector A Channel 2 254nm

Peak#	Ret. Time	Height	Area	Area%
1	5,410	1982214	8567701	100,000
Total		1982214	8567701	100,000
